# Copper-catalyzed stereoselective conjugate addition of alkylboranes to alkynoates

**DOI:** 10.3762/bjoc.11.265

**Published:** 2015-12-04

**Authors:** Takamichi Wakamatsu, Kazunori Nagao, Hirohisa Ohmiya, Masaya Sawamura

**Affiliations:** 1 Department of Chemistry, Faculty of Science, Hokkaido University, Sapporo 060-0810, Japan

**Keywords:** alkylborane, alkynoate, conjugate addition, copper, multisubstituted alkene

## Abstract

A copper-catalyzed conjugate addition of alkylboron compounds (alkyl-9-BBN, prepared by hydroboration of alkenes with 9-BBN-H) to alkynoates to form β-disubstituted acrylates is reported. The addition occurred in a formal *syn*-hydroalkylation mode. The *syn* stereoselectivity was excellent regardless of the substrate structure. A variety of functional groups were compatible with the conjugate addition.

## Introduction

Copper-mediated conjugate additions of organometallic reagents to alkynoates are powerful tools for the synthesis of multisubstituted alkenes [1–8]. Because of their broad availability and their compatibility with a multitude of functional groups, organoboron compounds are especially popular organometallic reagents. Recently, Yamamoto and co-workers developed copper-catalyzed conjugate additions of aryl- and allylboron compounds to alkynoates [9–10], but alkylboron compounds have not been used for these methods [11].

As related studies we reported earlier the copper-catalyzed conjugate addition of alkylboranes (alkyl-9-BBN) to imidazole-2-yl α,β-unsaturated ketones [12–14] and the copper-catalyzed three-component coupling with alkylboranes, alkynoates, and tributyltin methoxide to form trisubstituted alkenylstannanes [15]. The latter reaction pathway involved Sn-trapping of an alkenylcopper intermediate that was formed through *syn*-carbocupration of an alkylcopper(I) species across the C–C triple bond of the alkynoate. We envisioned that 1,2-hydroalkylation of the alkynoates might be possible through proton-trapping of an alkenylcopper intermediate.

Herein, we report a copper-catalyzed conjugate addition of alkylboranes to alkynoates, providing a versatile approach to β-disubstituted acrylates [16–19]. The addition occurred in a formal *syn*-hydroalkylation mode. The *syn* stereoselectivity was excellent regardless of the substrate structure, and a variety of functional groups were tolerated in both the alkylborane and the alkynoate.

## Results and Discussion

**Scheme 1:**
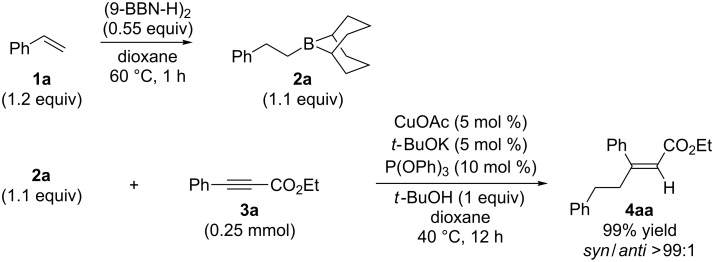
Conjugate addition of alkylborane **2a** to alkynoate **3a**.

Alkylborane **2a** (0.275 mmol), which was obtained via hydroboration of styrene (**1a**) with the 9-borabicyclo[3.3.1]nonane (9-BBN-H) dimer, and ethyl 3-phenylpropiolate (**3a**, 0.25 mmol) were treated with CuOAc (5 mol %), *t-*BuOK (5 mol %), P(OPh)_3_ (10 mol %), and *t-*BuOH (0.25 mmol) in 1,4-dioxane (1.2 mL) at 40 °C for 12 h. The reaction afforded a formal hydroalkylation product, β-disubstituted acrylate **4aa** in 99% isolated yield with >99% *syn* selectivity (Scheme 1).

The results of ligand screening for the reaction between **2a** and **3a** are summarized in Table 1. P(OPh)_3_ was the most effective ligand in terms of product yield and *syn* selectivity (Table 1, entry 1). The use of other monophosphine ligands such as PPh_3_ and PCy_3_ or the DPPE bisphopshine was also effective in promotion of the reaction, but resulted in a reduced stereoselectivity (Table 1, entries 2–4). No reaction occurred with *N*-heterocyclic carbenes (NHC) such as IMes or IPr (Table 1, entries 5 and 6). The reaction with (IMes)CuCl or (IPr)CuCl complex delivered no reaction product (data not shown). The reaction without a ligand resulted in a significantly decreased product yield while the *syn* selectivity was fairly high (Table 1, entry 7).

**Table 1:** Ligand effects.

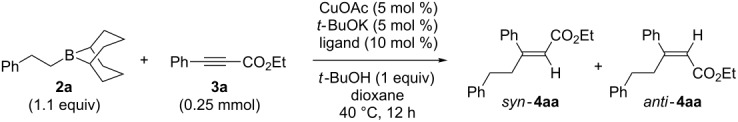			

Entry	Ligand^a^	Yield [%]^b^	*syn*/*anti*^c^

1	P(OPh)_3_	99	>99:1
2	PPh_3_	99	67:33
3	PCy_3_	56	64:36
4	DPPE	99	83:17
5	IMes	0	–
6	IPr	0	–
7	none	37	97:3

^a^IMes: 1,3-bis(2,4,6-trimethylphenyl)imidazole-2-ylidene, IPr: 1,3-bis(2,6-diisopropylphenyl)imidazole-2-ylidene. ^b^Yield determined by ^1^H NMR. ^c^Determined by ^1^H NMR or GC analysis of the crude product.

The use of less expensive CuCl as a copper salt was also effective to produce **4aa** in 90% yield with 99% *syn* selectivity. The reaction using MeOH as a proton source instead of *t*-BuOH caused a drastic reduction in the product yield with the syn selectivity slightly decreased (38%, *syn*/*anti* 97:3). The reduction of the yield might be due to the protonation of an alkylcopper species by the more acidic MeOH (vide infra). There was no reaction in the absence of a proton sourse. No hydroalkylation product at all could be found when alkyl-9-BBN **2a** was replaced by (2-phenylethyl)boronic acid pinacolate ester; the substrates hardly reacted at all.

A variety of β-disubstituted acrylates were accessible through the hydroboration–conjugate addition one-pot protocol with excellent *syn* stereoselectivities (Table 2). This protocol tolerated functional groups such as methoxy, ester, phthalimide, fluoro, cyano and aldehyde moieties in the alkylboranes and alkynoates (Table 2, entries 1–3, 6–9 and 11).

**Table 2:** Copper-catalyzed conjugate addition of alkylboranes **2** to alkynoates 3.^a^

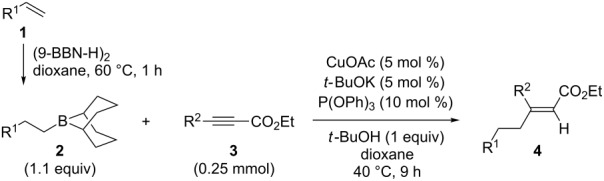					

Entry	Alkene	Alkynoate	Product	Yield [%]^b^	*syn*/*anti*^c^

1	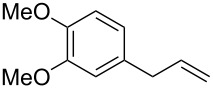 **1b**	**3a**	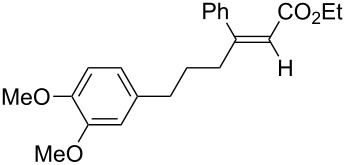 **4ba**	94	99:1
2	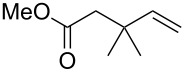 **1c**	**3a**	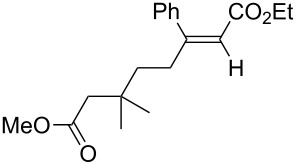 **4ca**	99	>99:1
3	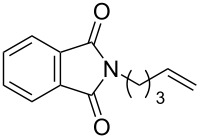 **1d**	**3a**	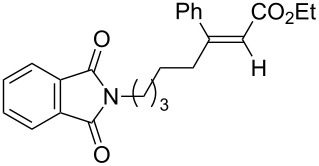 **4da**	87	99:1
4^d^	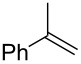 **1e**	**3a**	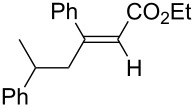 **4ea**	86	>99:1
5	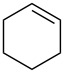 **1f**	**3a**	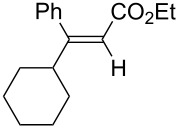 **4fa**	0	–
6	**1a**	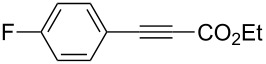 **3b**	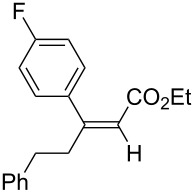 **4ab**	96	98:2
7	**1a**	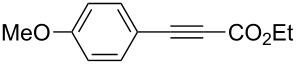 **3c**	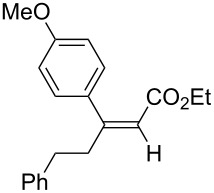 **4ac**	91	99:1
8	**1a**	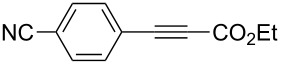 **3d**	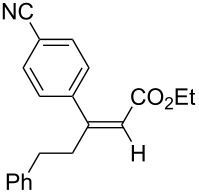 **4ad**	88	99:1
9	**1a**	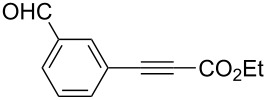 **3e**	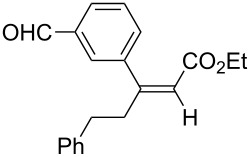 **4ae**	93	99:1
10	**1a**	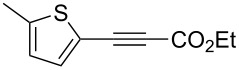 **3f**	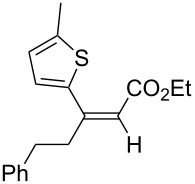 **4af**	95	94:6
11	**1a**	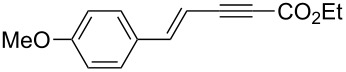 **3g**	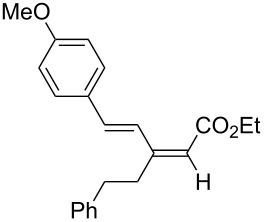 **4ag**	90	>99:1
12	**1a**	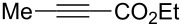 **3h**	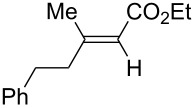 **4ah**	94	>99:1
13	**1a**	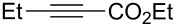 **3i**	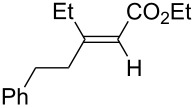 **4ai**	98	>99:1
14	**1a**	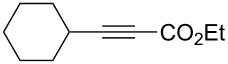 **3j**	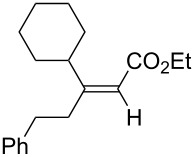 **4aj**	93	>99:1

^a^The reaction was carried out with **3** (0.25 mmol), **2** (0.275 mmol), CuOAc (5 mol %), *t*-BuOK (5 mol %), P(OPh)_3_ (10 mol %) and *t*-BuOH (0.25 mmol) in dioxane (1.2 mL) at 40 °C for 12 h. Alkylborane **2** was prepared in advance by hydroboration of **1** with the 9-BBN-H dimer at 60 °C for 1 h and used without purification. ^b^Yield of isolated product. ^c^Determined by ^1^H NMR or GC analysis of the crude product. ^d^Diasteremeric ratio (1:1).

The data in Table 2 show the variety of functional groups attached to alkylboranes **2** that are tolerated in the reaction. The rather crowded alkylborane **2c**, which was prepared from tertiary alkyl substituted terminal alkene **1c**, reacted nicely (Table 2, entry 2). β-Branched alkylborane **2e**, prepared from α-methylstyrene (**1e**), provided **4ea** in good yield (Table 2, entry 4). Unfortunately, however, the reaction of secondary alkylboranes made from internal alkenes, did not work (Table 2, entry 5).

The variety of alkynoates used is also shown in Table 2. The fluoro atom and the methoxy, cyano and aldehyde groups were acceptable as *para* or *meta*-substituents on the aromatic ring at the β-positions (Table 2, entries 6–9). The alkynoate **3f** bearing a 2-thienyl group at the β-position is also compatible with the conjugate addition and gave 94% *syn* selectivity (Table 2, entry 10). The 1,3-enyne derivative **3g** reacted regioselectively to afford a conjugated 2,4-dienoate **4ag** in 90% yield with excellent *syn* selectivity (Table 2, entry 11).

Alkyl groups were also acceptable as β-substituent of the alkynoates (Table 2, entries 12–14). Alkynoate **3h** with a methyl group at the β-position reacted with an excellent stereoselectivity (Table 2, entry 12). The alkynoates with an ethyl (**3i**) or cyclohexyl group (**3j**) were also suitable substrates (Table 2, entries 13 and 14).

**Scheme 2:**
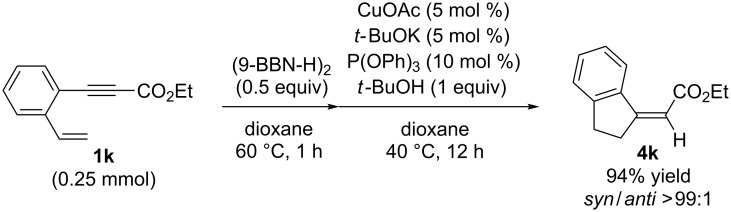
Synthesis of five membered carbocycle.

Alkene hydroboration of **1k** followed by copper-catalyzed intramolecular conjugate addition enabled the formation of the corresponding five-membered carbocycle **4k** in 94% yield with complete syn selectivity (Scheme 2).

**Scheme 3:**
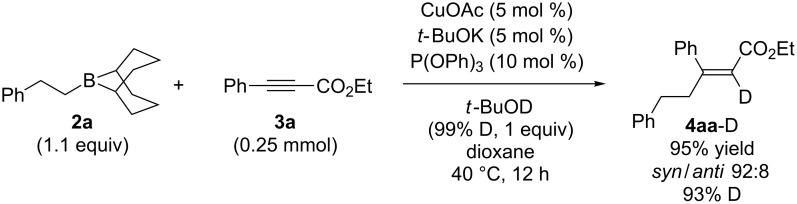
Deuterium-labeling experiment.

To gain insight into the mechanism of the copper-catalyzed conjugate addition, the reaction between **2a** and **3a** with *t*-BuOD under the optimum conditions was conducted (Scheme 3). The reaction afforded **4aa**-D, which is deuterated at the α-position of the carbonyl group (93% D). The *syn* selectivity was slightly decreased due to the deuterium isotope effect: Slower D-trap caused isomerization of organocopper intermediates (vide infra). This experimental result indicates that *t*-BuOH acts as a proton source.

**Figure 1:**
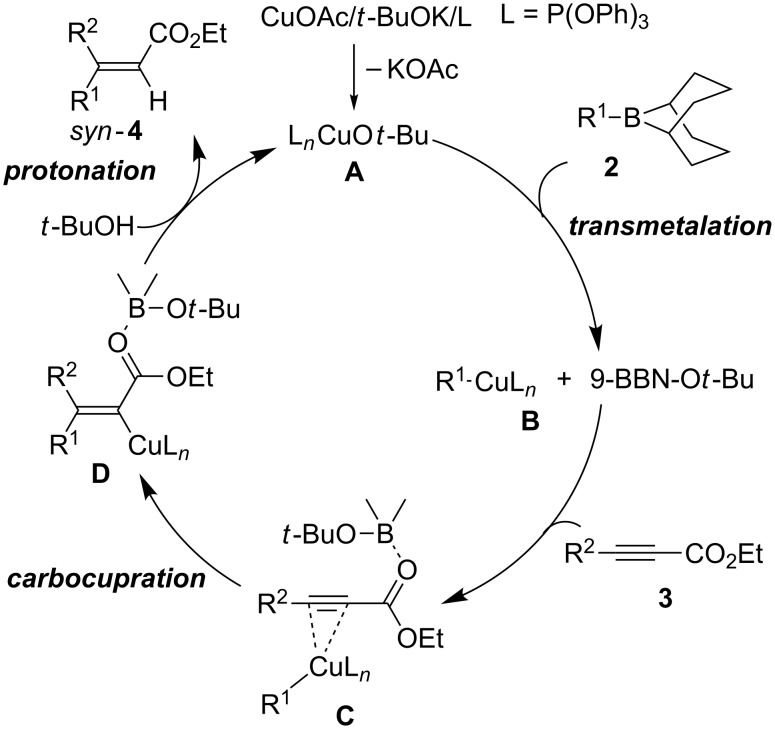
Possible mechanism.

A possible mechanism for the present copper catalysis is proposed in Figure 1. An alkoxycopper complex (**A**) is initially formed by the reaction of CuOAc, *t*-BuOK and P(OPh)_3_. Boron-to-cupper transmetalation between **A** and the alkylborane **2** occurs to form an alkylcopper(I) species (**B**) and a *t*-butoxyborane (9-BBN-O*t*-Bu) [12–15,20–25]. Subsequently, the alkylcopper species **B** forms a π-complex (**C**) with alkynoate **3**. Then, *syn*-carbocupration across the C–C triple bond of **C** with the assistance of Lewis acidic activation with the *tert*-butoxyborane gave an alkenylcopper intermediate (**D**). Finally, protonolysis by *t*-BuOH produces *syn*-**4**, regenerating the alkoxycopper complex **A**.

**Figure 2:**
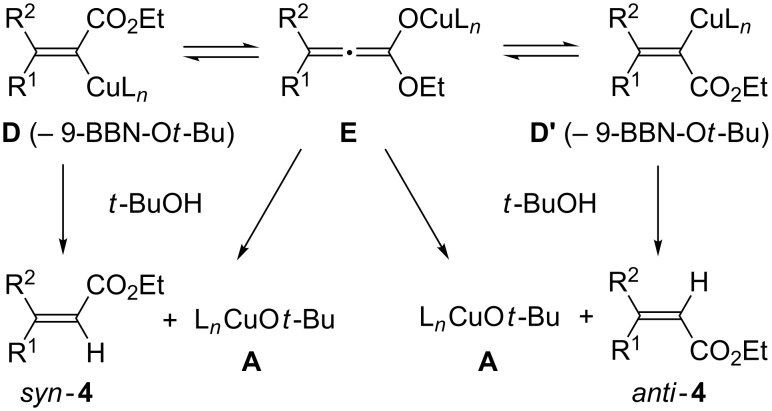
Isomerization of the alkenylcopper intermediates.

The minor occurrence of *anti*-addition is likely due to the geometrical isomerization of the alkenylcopper species (**D**/**D'**) through a copper(I) allenoate complex (**E**, Figure 2) [15,26]. The resulting allenoate **E** can undergo protonolysis to form either *syn*-**4** or *anti*-**4** depending on the substituent effects of R^1^ and R^2^, while the isomerized alkenylcopper(I) **D'** should preferentially yield *anti*-**4**. The reduction of *syn* selectivity in the reaction with PPh_3_, PCy_3_ and DPPE may also be due to this isomerization (Table 1, entries 2–4).

## Conclusion

In summary, a copper-catalyzed conjugate addition of alkylboranes (alkyl-9-BBN) to alkynoates to form β-disubstituted acrylates is reported. The addition occurred in a formal *syn*-hydroalkylation mode. The stereoselectivity was excellent regardless of the substrate structure. The availability of alkylboranes through in situ alkene hydroboration is an attractive feature of this protocol and various functional groups are tolerated in both the alkylborane and alkynoate substrates.

## Experimental

The reaction shown in Scheme 1 was conducted in a similar manner as described before [15]. Styrene (**1a**, 33 μL, 0.289 mmol) and (9-BBN-H)_2_ (33.6 mg, 0.138 mmol) were placed in a vial containing a magnetic stirring bar. The vial was sealed with a Teflon^®^-coated silicon rubber septum, and the vial was evacuated and filled with argon. 1,4-Dioxane (0.4 mL) was added to the vial, and the mixture was stirred at 60 °C for 1 h to prepare an alkylborane **2a**. Meanwhile, CuOAc (1.5 mg, 0.0125 mmol), P(OPh)_3_ (6.9 μL, 0.025 mmol) and *t*-BuOK (1.4 mg, 0.0125 mmol) were placed in another vial. The vial was sealed with a Teflon^®^-coated silicon rubber septum, evacuated, and then filled with argon. After 1,4-dioxane (0.6 mL) was added to the vial, the mixture was stirred at 25 °C for 1 h. Next, the alkylborane solution was transferred to the vial containing the Cu(I) complex, followed by the addition of alkynoate **3a** (41.3 μL, 0.25 mmol) and *t*-BuOH (24 μL, 0.25 mmol). After 12 h stirring at 40 °C, diethyl ether was added to the mixture. The mixture was filtered through a short plug of silica gel, which was then washed with diethyl ether. After the solvent was removed under reduced pressure, flash chromatography on silica gel (0–5% EtOAc/hexane) provided **4aa** (69.4 mg, 0.248 mmol) in 99% yield with >99:1 *syn*/*anti* selectivity.

## Supporting Information

File 1 Experimental procedures, spectroscopic and analytical data, and copies of NMR spectra for newly synthesized compounds.
