# Supplementary material for: Copper-catalyzed stereoselective conjugate addition of alkylboranes to alkynoates
Source: Beilstein J Org Chem. 2015 Dec 4;11:2444–50. doi: 10.3762/bjoc.11.265 (PMC4685882; doi:10.3762/bjoc.11.265)
Supplement: File 1 — Experimental procedures, spectroscopic and analytical data, and copies of NMR spectra for newly synthesized compounds. [file Beilstein_J_Org_Chem-11-2444-s001.pdf]

Supporting Information

for

Copper-catalyzed stereoselective conjugate addition of alkylboranes to alkynoates

Takamichi Wakamatsu, Kazunori Nagao, Hirohisa Ohmiya* and Masaya Sawamura*

Address: Department of Chemistry, Faculty of Science, Hokkaido University, Sapporo 060-0810, Japan

Email: Hirohisa Ohmiya - ohmiya@sci.hokudai.ac.jp; Masaya Sawamura - sawamura@sci.hokudai.ac.jp

* Corresponding author

Experimental procedures, spectroscopic and analytical data, and copies of NMR spectra for newly synthesized compounds

Table of Contents

Instrumentation and chemicals	S1
Procedures for copper-catalyzed conjugate addition	S2
Characterization data for β-disubstituted acrylates	S3–S7
References	S7
NMR spectra	S8–S38

Instrumentation and chemicals

NMR spectra were recorded on a JEOL ECX-400, operating at 400 MHz for ^{1}H NMR, 100.5 MHz for ^{13}C NMR. Chemical shift values for ^{1}H and ^{13}C are referenced to Me_4Si. Chemical shifts are reported in δ ppm. Mass spectra were obtained with Thermo Fisher Scientific Exactive, JEOL JMS-T100LP or JEOL JMS-700TZ at the Instrumental Analysis Division, Equipment Management Center, Creative Research Institution, Hokkaido University. Elemental analysis was performed at the Instrumental Analysis Division, Equipment Management Center, Creative Research Institution, Hokkaido University. TLC analyses were performed on commercial glass plates bearing 0.25-mm layer of Merck Silica gel $60F_{254}$. Silica gel (Kanto Chemical Co., Silica gel 60 N, spherical, neutral) was used for column chromatography. Melting points were measured on a Yanaco MP-500D apparatus.

All reactions were carried out under nitrogen or argon atmosphere. Materials were obtained from commercial suppliers or prepared according to standard procedures unless otherwise noted. 9-Borabicyclo[3,3,1]nonane dimer $(9\text{-BBN-H})_2$ was purchased from Kanto Chemical Co., stored

under nitrogen, and used as is. 1,4-Dioxane was purchased from Kanto Chemical Co. and stored under argon.

Alkenes **1a–c** and **1e** were purchased from TCI Chemical Co., stored under nitrogen, and used as it is. Alkenes **1d** and **1k** are known compounds.[1] Alkynoates **3a**, **3h** and **3i** were purchased from Aldrich Chemical Co., distilled and stored under nitrogen. Alkynoates **3b**[1], **3c**[1], **3f**[1], **3g**[1] and **3j**[2] are known compounds. Alkynoates **3d**[3] and **3e**[4] were prepared by Sonogashira coupling between the corresponding aryl iodides and ethyl propiolate.[3] Alkynoate **3g** was prepared by the reaction between the corresponding lithium acetylide and ethyl chloroformate[5].

Procedures for copper-catalyzed conjugate addition

Typical procedure for copper-catalyzed stereoselective conjugate addition of alkylboranes to alkynoates. The reaction in Scheme 1 was conducted in a similar manner as described before ref. 1. Styrene (**1a**) (33 µL, 0.289 mmol) and $(9\text{-BBN-H})_2$ (33.6 mg, 0.138 mmol) were placed in a vial containing a magnetic stirring bar. The vial was sealed with a Teflon®-coated silicon rubber septum, and the vial was evacuated and filled with argon. 1,4-Dioxane (0.4 mL) was added to the vial, and the mixture was stirred at 60 °C for 1 h to prepare an alkylborane **2a**. Meanwhile, CuOAc (1.5 mg, 0.0125 mmol), $P(OPh)_3$ (6.9 µL, 0.025 mmol) and *t*-BuOK (1.4 mg, 0.0125 mmol) were placed in another vial. The vial was sealed with a Teflon®-coated silicon rubber septum, evacuated, and then filled with argon. After 1,4-Dioxane (0.6 mL) was added to the vial, the mixture was stirred at 25 °C for 1 h. Next, the alkylborane solution was transferred to the vial containing the Cu(I) complex. Next, alkynoate **3a** (41.3 µL, 0.25 mmol) and *t*-BuOH (24 µL, 0.25 mmol) were added. After 12 h stirring at 40 °C, diethyl ether was added to the mixture. The mixture was filtered through a short plug of silica gel, which was then washed with diethyl ether. After the solvent was removed under reduced pressure, flash chromatography on silica gel (0–5% EtOAc/hexane) provided **4aa** (69.4 mg, 0.248 mmol) in 99% yield with >99:1 *syn*/*anti* selectivity.

Procedure for the synthesis of 4k (Scheme 2). 1k (50 mg, 0.25 mmol) and $(9\text{-BBN-H})_2$ (30.5 mg, 0.125 mmol) were placed in a vial containing a magnetic stirring bar. The vial was sealed with a Teflon®-coated silicon rubber septum and the vial was evacuated and filled with argon. 1,4-Dioxane (0.4 mL) was added to the vial, and then the mixture was stirred at 60 °C for 1 h to prepare the alkylborane. On the other hand, CuOAc (1.5 mg, 0.0125 mmol), $P(OPh)_3$ (6.9 µL, 0.025 mmol) and *t*-BuOK (1.4 mg, 0.0125 mmol) were placed in another vial. The vial was sealed with a Teflon®-coated silicon rubber septum and the vial was evacuated and filled with argon. After 1,4-dioxane (0.6 mL) was added to the vial, the mixture was stirred at 25 °C for 1 h. Next, the alkylborane solution was transferred to the vial containing a Cu(I) complex. Then, *t*-BuOH (24 µL, 0.25 mmol) was added. After 12 h stirring at 40 °C, diethyl ether was added to the mixture. The mixture was filtered through a short plug of silica gel, which was then washed with diethyl ether. After the solvent was removed under reduced pressure, flash chromatography on silica gel (0–5% EtOAc/hexane) provided **4k** (47.5 mg, 0.238 mmol) in 94% yield with >99:1 *syn*/*anti* selectivity.

Characterization data for β-disubstituted acrylates

(*Z*)-Ethyl 3,5-diphenyl-2-pentenoate (4aa)

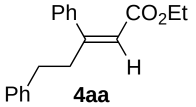

4aa

The product **4aa** was purified by flash chromatography on silica gel (0–5% EtOAc/hexane). Colorless Oil. ^{1}H NMR (400 MHz, $CDCl_3$) δ 1.06 (t, J = 7.2 Hz, 3H), 2.71–2.76 (m, 4H), 3.98 (q, J = 7.2 Hz, 2H), 5.90 (d, J = 1.2 Hz, 1H), 7.12–7.15 (m, 2H), 7.18–7.20 (m, 3H). 7.25–7.30 (m, 2H), 7.31–7.37 (m, 3H). ^{13}C NMR (100.5 MHz, $CDCl_3$) δ 13.88, 33.72, 42.01, 59.77, 117.65, 126.06, 127.15, 127.69, 127.88, 128.24, 128.38, 139.78, 140.81, 158.44, 165.92. HRMS–ESI (*m/z*): $[M+Na]^+$ calcd for $C_{19}H_{20}O_2Na$, 303.13555; found, 303.13560. The stereochemistry was determined by NOESY experiments.

(*Z*)-Ethyl 6-(3,4-dimethoxyphenyl)-3-phenyl-2-hexenoate (4ba)

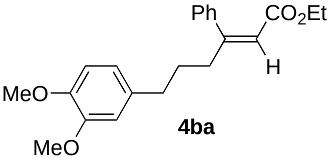

4ba

The product **4ba** was purified by flash chromatography on silica gel (0–20% EtOAc/hexane). Colorless Oil. ^{1}H NMR (400 MHz, $CDCl_3$) δ 1.06 (t, J = 7.2 Hz, 3H), 1.70 (quin, J = 7.6 Hz, 2H), 2.47 (td, J = 7.6, 0.8 Hz, 2H), 2.56 (t, J = 7.6 Hz, 2H), 3.84 (s, 3H), 3.85 (s, 3H), 3.98 (q, J = 7.2 Hz, 2H), 5.89 (t, J = 0.8 Hz, 1H), 6.62–6.67 (m, 2H), 6.77 (m, 1H), 7.14–7.17 (m, 2H). 7.28–7.37 (m, 3H). ^{13}C NMR (100.5 MHz, $CDCl_3$) δ 13.83, 28.99, 34.60, 39.59, 55.65, 55.79, 59.68, 111.04, 111.51, 117.48, 120.07, 127.00, 127.51, 127.76, 134.16, 139.90, 147.06, 148.65, 159.11, 165.89. HRMS–ESI (*m/z*): $[M+Na]^+$ calcd for $C_{22}H_{26}O_4Na$, 377.17233; found, 377.17247. The stereochemistry was assigned by consideration of the reaction pathway.

(*Z*)-1-Ethyl 8-methyl 6,6-dimethyl-3-phenyl-2-octenedioate (4ca)

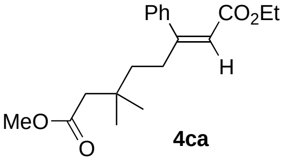

4ca

The product **4ca** was purified by flash chromatography on silica gel (0–8% EtOAc/hexane). Colorless Oil. ^{1}H NMR (400 MHz, $CDCl_3$) δ 0.97 (s, 6H), 1.07 (t, J = 7.6 Hz, 3H), 1.40–1.44 (m, 2H), 2.18 (s, 2H), 2.41–2.45 (m, 2H), 3.59 (s, 3H), 3.98 (q, J = 7.6 Hz, 2H), 5.89 (s, 1H), 7.14–7.17 (m, 2H), 7.28–7.35 (m, 3H). ^{13}C NMR (100.5 MHz, $CDCl_3$) δ 13.87, 27.17, 33.03, 35.10, 39.69, 45.42, 51.09, 59.68, 117.04, 126.98, 127.53, 127.77, 140.16, 159.72, 165.96, 172.28. HRMS–ESI (*m/z*): $[M+Na]^+$ calcd for $C_{19}H_{26}O_4Na$, 341.17233; found, 341.17238. The stereochemistry was assigned by consideration of the reaction pathway.

(*Z*)-Ethyl 8-(1,3-dioxoisoindolin-2-yl)-3-phenyl-2-octenoate (4da)

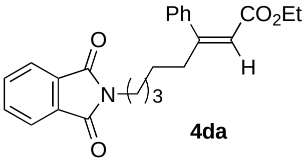

4da

The product **4da** was purified by flash chromatography on silica gel (0–20% EtOAc/hexane). White Solid. M.p. 63–65 °C. ^{1}H NMR (400 MHz, $CDCl_3$) δ 1.07 (t, J = 7.2 Hz, 3H), 1.30–1.50 (m, 4H), 1.62 (quin, J = 7.6 Hz, 2H), 2.43 (td, J = 7.6, 1.2 Hz, 2H), 3.65 (t, J = 7.6 Hz, 2H), 3.97 (q, J = 7.2 Hz, 2H), 5.86 (s, 1H), 7.12–7.14 (m, 2H), 7.24–7.35 (m, 3H), 7.68–7.73 (m, 2H), 7.81–7.85 (m, 2H). ^{13}C NMR (100.5 MHz, $CDCl_3$) δ 13.82, 26.20, 26.77, 28.15, 37.66, 40.04, 59.62, 117.69, 123.03, 126.91, 127.44, 127.71, 131.95, 133.77, 139.91, 159.23, 165.86, 168.26. HRMS–ESI (*m/z*): $[M+Na]^{+}$ calcd for $C_{24}H_{25}O_4NNa$, 414.16758; found, 414.16776. The stereochemistry was assigned by consideration of the reaction pathway.

(*Z*)-Ethyl 3,5-diphenyl-2-hexenoate (4ea)

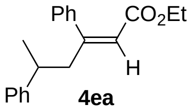

4ea

The product **4ea** was purified by flash chromatography on silica gel (0–5% EtOAc/hexane). White Solid. M.p. 61–62 °C. ^{1}H NMR (400 MHz, $CDCl_3$) δ 1.04 (t, J = 7.2 Hz, 3H), 1.23 (d, J = 6.4 Hz, 3H), 2.61–2.75 (m, 2H), 2.82 (m, 1H), 3.95 (q, J = 7.2 Hz, 2H), 5.81 (s, 1H), 7.09–7.21 (m, 5H), 7.26–7.38 (m, 5H). ^{13}C NMR (100.5 MHz, $CDCl_3$) δ 13.86, 21.53, 37.26, 49.03, 59.73, 118.93, 126.17, 126.83, 127.31, 127.72, 127.81, 128.38, 139.46, 146.13, 157.45, 165.78. HRMS–ESI (*m/z*): $[M+Na]^{+}$ calcd for $C_{20}H_{22}O_2Na$, 317.15120; found, 317.15142. The stereochemistry was assigned by consideration of the reaction pathway.

(*Z*)-Ethyl 3-(4-fluorophenyl)-5-phenyl-2-pentenoate (4ab)

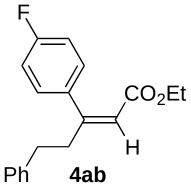

4ab

The product **4ab** was purified by flash chromatography on silica gel (0–5% EtOAc/hexane). Colorless Oil. ^{1}H NMR (400 MHz, $CDCl_3$) δ 1.10 (t, J = 7.2 Hz, 3H), 2.67–2.76 (m, 4H), 4.00 (q, J = 7.2 Hz, 2H), 5.90 (s, 1H), 7.03–7.09 (m, 2H), 7.10–7.21 (m, 5H), 7.25–7.31 (m, 2H). ^{13}C NMR (100.5 MHz, $CDCl_3$) δ 13.93, 33.71, 42.03, 59.86, 114.89 (d, $J_{C–F}$ = 21 Hz), 118.04, 126.12, 128.22, 128.42, 129.04 (d, $J_{C–F}$ = 8.6 Hz), 135.47 (d, $J_{C–F}$ = 3.8 Hz), 140.60, 157.39, 162.33 (d, $J_{C–F}$ = 246 Hz), 165.75. HRMS–ESI (*m/z*): $[M+Na]^{+}$ calcd for $C_{19}H_{19}O_2FNa$, 321.12613; found, 321.12632. The stereochemistry was assigned by consideration of the reaction pathway.

(*Z*)-Ethyl 3-(4-methoxyphenyl)-5-phenyl-2-pentenoate (4ac)

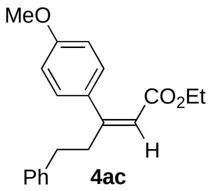

4ac

The product **4ac** was purified by flash chromatography on silica gel (0–5% EtOAc/hexane). Colorless Oil. ^{1}H NMR (400 MHz, $CDCl_3$) δ 1.12 (t, *J* = 7.2 Hz, 3H), 2.68–2.76 (m, 4H), 3.83 (s, 3H), 4.01 (q, *J* = 7.2 Hz, 2H), 5.85 (s, 1H), 6.89–6.92 (m, 2H), 7.11–7.25 (m, 4H), 7.25–7.27 (m, 3H). ^{13}C NMR (100.5 MHz, $CDCl_3$) δ 14.00, 33.94, 42.06, 55.11, 59.70, 113.25, 117.06, 126.00, 128.23, 128.34, 128.75, 131.49, 140.89, 158.12, 159.29, 166.07. HRMS–ESI (*m/z*): $[M+Na]^+$ calcd for $C_{20}H_{22}O_3Na$, 333.14612; found, 333.14600. The stereochemistry was assigned by consideration of the reaction pathway.

(*Z*)-Ethyl 3-(4-cyanophenyl)-5-phenyl-2-pentenoate (4ad)

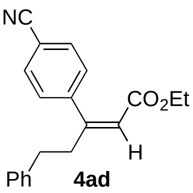

4ad

The product **4ad** was purified by flash chromatography on silica gel (0–10% EtOAc/hexane). Colorless Oil. ^{1}H NMR (400 MHz, $CDCl_3$) δ 1.10 (t, *J* = 7.6 Hz, 3H), 2.68–2.77 (m, 4H), 3.99 (q, *J* = 7.6 Hz, 2H), 5.96 (s, 1H), 7.10–7.13 (m, 2H), 7.21 (m, 1H), 7.23–7.30 (m, 4H), 7.64–7.67 (m, 2H). ^{13}C NMR (100.5 MHz, $CDCl_3$) δ 13.89, 33.48, 41.46, 60.11, 111.38, 118.70, 118.93, 126.30, 128.00, 128.17, 128.50, 131.74, 140.06, 145.03, 156.62, 165.19. HRMS–ESI (*m/z*): $[M+Na]^+$ calcd for $C_{20}H_{19}O_2NNa$, 328.13080; found, 328.13101. The stereochemistry was assigned by consideration of the reaction pathway.

(*Z*)-Ethyl 3-(3-formylphenyl)-5-phenyl-2-pentenoate (4ae)

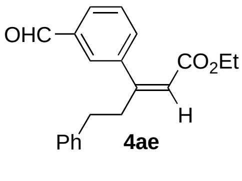

4ae

The product **4ad** was purified by flash chromatography on silica gel (0–15% EtOAc/hexane). Colorless Oil. ^{1}H NMR (400 MHz, $CDCl_3$) δ 1.08 (t, *J* = 7.2 Hz, 3H), 2.72–2.80 (m, 4H), 3.98 (q, *J* = 7.2 Hz, 2H), 5.97 (d, *J* = 1.6 Hz, 1H), 7.11–7.13 (m, 2H), 7.19 (m, 1H), 7.25–7.29 (m, 2H), 7.43 (m, 1H), 7.54 (m, 1H), 7.69 (m, 1H), 7.85 (m, 1H), 10.02 (s, 1H). ^{13}C NMR (100.5 MHz, $CDCl_3$) δ 13.88, 33.59, 41.73, 59.94, 118.59, 126.20, 128.19, 128.25, 128.44, 128.56, 129.13, 133.45, 136.06, 140.29, 140.85, 157.10, 165.40, 192.03. HRMS–ESI (*m/z*): $[M+Na]^+$ calcd for $C_{20}H_{20}O_3Na$, 331.13047; found, 331.13046. The stereochemistry was assigned by consideration of the reaction pathway.

(*Z*)-Ethyl 3-(5-methylthiophen-2-yl)-5-phenyl-2-pentenoate (4af)

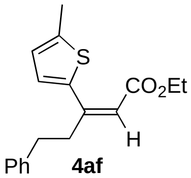

4af

The product **4af** was purified by flash chromatography on silica gel (0–5% EtOAc/hexane). Colorless Oil. ^{1}H NMR (400 MHz, $CDCl_3$) δ 1.23 (t, J = 7.2 Hz, 3H), 2.50 (d, J = 0.8 Hz, 3H), 2.72–2.83 (m, 4H), 4.13 (q, J = 7.2 Hz, 2H), 5.76 (s, 1H), 6.72 (m, 1H), 7.16–7.22 (m, 4H), 7.26–7.32 (m, 2H). ^{13}C NMR (100.5 MHz, $CDCl_3$) δ 14.08, 15.28, 35.17, 42.85, 59.95, 116.17, 125.26, 126.07, 128.28, 128.37, 129.22, 136.88, 140.85, 142.16, 148.52, 166.08. HRMS–ESI (*m/z*): $[M+Na]^+$ calcd for $C_{18}H_{20}O_2NaS$, 323.10762; found, 323.10757. The stereochemistry was assigned by consideration of the reaction pathway.

(2*Z*,4*E*)-Ethyl 5-(4-methoxyphenyl)-3-phenethyl-2,4-pentadienoate (4ag)

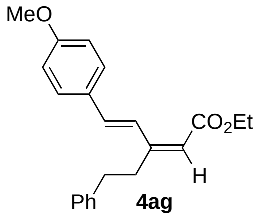

4ag

The product **4ag** was purified by flash chromatography on silica gel (0–5% EtOAc/hexane). Colorless Oil. ^{1}H NMR (400 MHz, $CDCl_3$) δ 1.32 (t, J = 7.2 Hz, 3H), 2.74–2.78 (m, 2H), 2.87–2.91 (m, 2H), 3.83 (s, 3H), 4.21 (q, J = 7.2 Hz, 2H), 5.70 (s, 1H), 6.87–6.91 (m, 2H), 6.94 (d, J = 16.4 Hz, 1H), 7.19–7.27 (m, 3H), 7.27–7.34 (m, 2H), 7.47–7.53 (m, 2H), 8.27 (d, J = 16.4 Hz, 1H). ^{13}C NMR (100.5 MHz, $CDCl_3$) δ 14.31, 35.63, 35.93, 55.25, 59.70, 114.10, 116.11, 122.80, 126.13, 128.27, 128.45, 128.74, 129.42, 134.38, 141.32, 154.41, 160.09, 166.64. HRMS–ESI (*m/z*): $[M+Na]^+$ calcd for $C_{22}H_{24}O_3Na$, 359.16177; found, 359.16168. The stereochemistry was assigned by consideration of the reaction pathway.

(*E*)-Ethyl 3-methyl-5-phenyl-2-pentenoate (4ah)

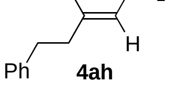

4ah

The product **4ah** was purified by flash chromatography on silica gel (0–3% EtOAc/hexane). **4ah** was consistent with the the literature data.[6]

(*E*)-Ethyl 3-ethyl-5-phenyl-2-pentenoate (4ai)

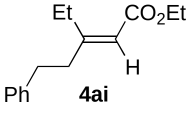

4ai

The product **4ai** was purified by flash chromatography on silica gel (0–5% EtOAc/hexane). Colorless Oil. ^{1}H NMR (400 MHz, $CDCl_3$) δ 1.10 (t, *J* = 7.6 Hz, 3H), 1.28 (t, *J* = 6.8 Hz, 3H), 2.43–2.48 (m, 2H), 2.68 (q, *J* = 6.8 Hz, 2H), 2.76–2.80 (m, 2H), 4.15 (q, *J* = 7.6 Hz, 2H), 5.66 (s, 1H), 7.12–7.32 (m, 5H). ^{13}C NMR (100.5 MHz, $CDCl_3$) δ 12.95, 14.25, 25.39, 34.08, 39.69, 59.49, 115.10, 126.05, 128.22, 128.42, 141.18, 164.76, 166.37. HRMS–ESI (*m/z*): $[M+H]^+$ calcd for $C_{15}H_{21}O_2$, 233.15361; found, 233.15363. The stereochemistry was assigned by consideration of the reaction pathway.

(*Z*)-Ethyl 3-cyclohexyl-5-phenyl-2-pentenoate (4aj)

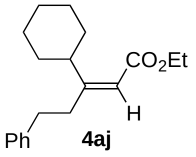

4aj

The product **4aj** was purified by flash chromatography on silica gel (0–5% EtOAc/hexane). Colorless Oil. ^{1}H NMR (400 MHz, $CDCl_3$) δ 1.15 (t, *J* = 7.2Hz, 3H), 1.30–1.45 (m, 4H), 1.60–1.80 (m, 5H), 2.39–2.45 (m, 2H), 2.74–2.78 (m, 2H), 3.69 (m, 1H), 4.16 (q, *J* = 7.2 Hz, 2H), 5.66 (s, 1H), 7.19–7.32 (m, 5H). ^{13}C NMR (100.5 MHz, $CDCl_3$) δ 14.30, 26.10, 26.33, 30.96, 34.65, 34.95, 40.55, 59.50, 114.34, 126.05, 128.24, 128.45, 141.52, 166.54, 167.68. HRMS–ESI (*m/z*): $[M+Na]^+$ calcd for $C_{19}H_{26}O_2Na$, 309.18250; found, 309.18271. The stereochemistry was assigned by consideration of the reaction pathway.

(*Z*)-Ethyl 2-(2,3-dihydro-1*H*-inden-1-ylidene)acetate (4k)

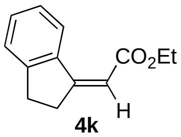

4k

The product **4k** was purified by flash chromatography on silica gel (0–5% EtOAc/hexane). Colorless Oil. ^{1}H NMR (400 MHz, $CDCl_3$) δ 1.32 (t, *J* = 7.2 Hz, 3H), 2.90–2.94 (m, 2H), 2.98–3.01 (m, 2H), 4.22 (q, *J* = 7.2 Hz, 2H), 5.97 (t, *J* = 2.0 Hz, 1H), 7.26–7.35 (m, 3H), 8.82 (d, *J* = 7.6 Hz, 1H). ^{13}C NMR (100.5 MHz, $CDCl_3$) δ 14.33, 29.54, 35.61, 59.78, 110.81, 124.97, 126.53, 128.68, 130.70, 137.52, 150.90, 160.49, 166.28. HRMS–ESI (*m/z*): $[M+Na]^+$ calcd for $C_{13}H_{14}O_2Na$, 225.08860; found, 225.08881. The stereochemistry was assigned by consideration of the reaction pathway.

References

1. Wakamatsu,T.; Nagao, K.; Ohmiya, H.; Sawamura, M. *Angew. Chem. Int. Ed.* **2013**, *52,* 11620.
2. Kwong, C. K-W.; Fu, M. Y.; Law, H. C-H.; Toy, P. H. *Synlett* **2010**, 2617.
3. Cai, S.; Yang, K.; Wang, D. Z. *Org. Lett.* **2014**, *16*, 2606.
4. Rao, H.; Fu, H.; Jiang, Y. Zhao, Y. *Adv. Synth. Catal.* **2010**, *352*, 458.
5. Nagao, K.; Ohmiya, H., Sawamura, M. *J. Am. Chem. Soc.* **2014**, *136*, 10605.
6. Miura, K.; Ebine, M.; Ootsuka, K.; Ichikawa, J.; Hosomi, A. *Chem. Lett.* **2009**, *38*, 832.

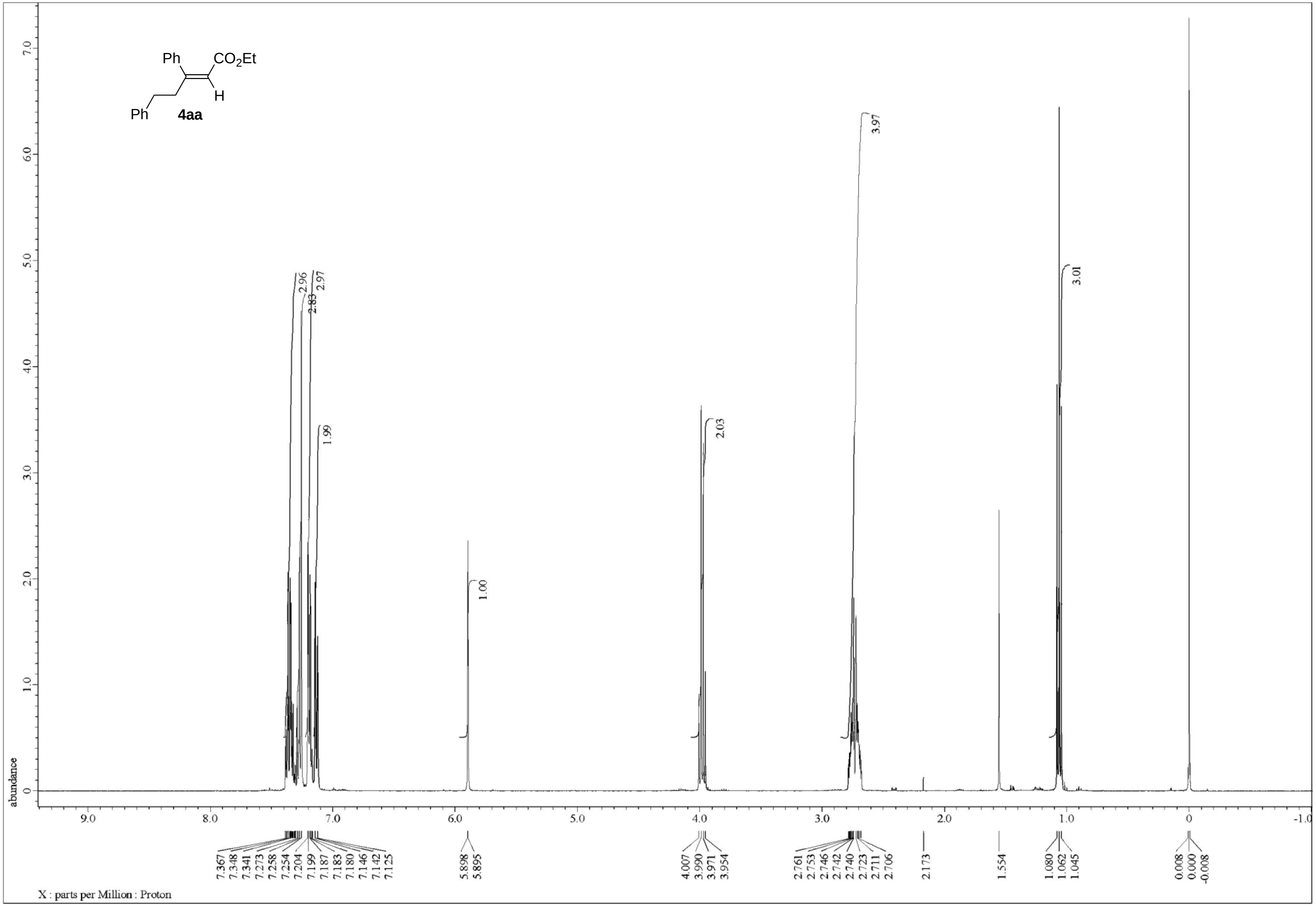
Ph
CO2Et
H
Ph
4aa
abundance
X : parts per Million : Proton

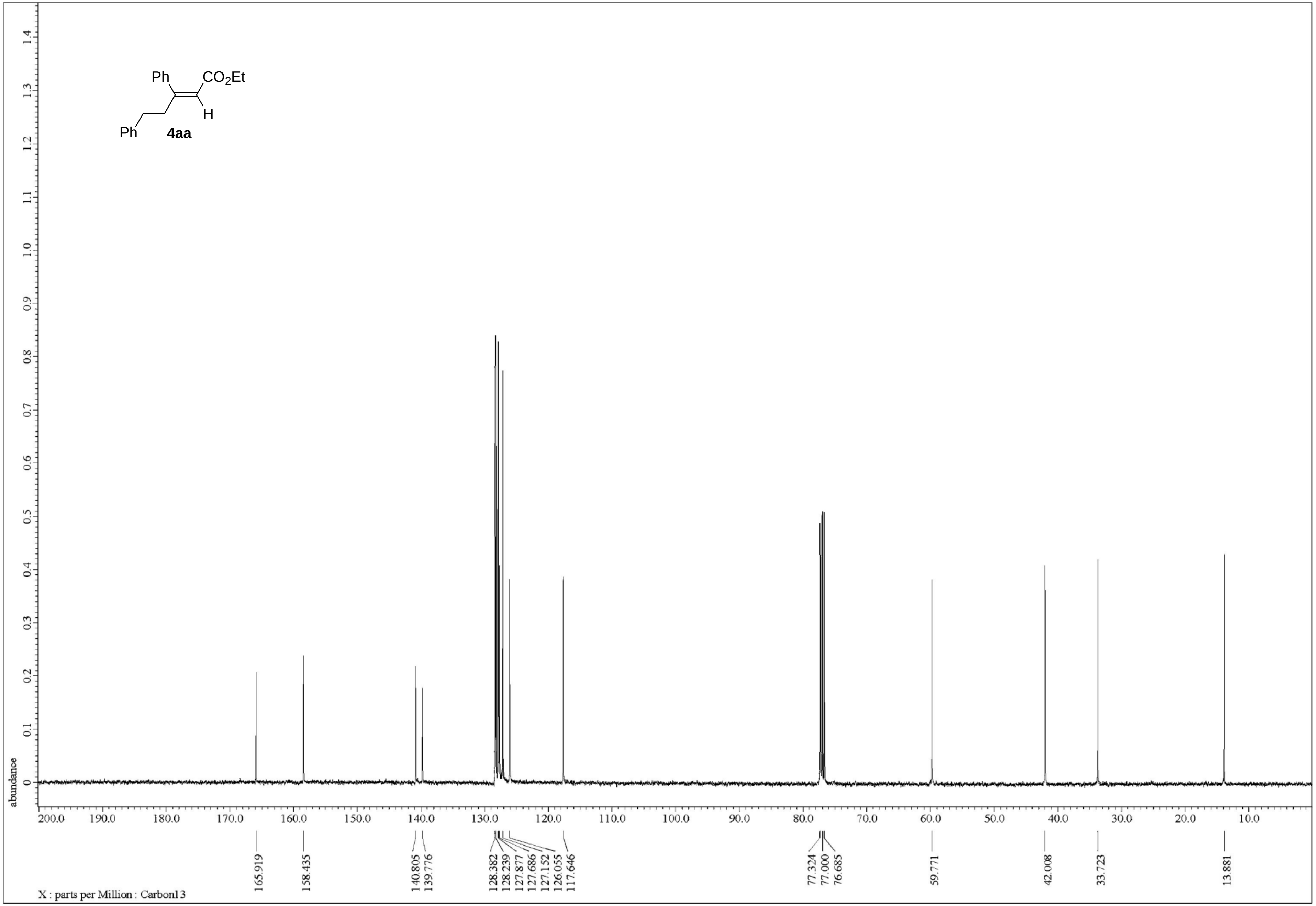
Ph
CO2Et
H
Ph
4aa
abundance
X : parts per Million : Carbon13
165.919
158.435
140.805
139.776
128.382
128.239
127.877
127.686
127.152
126.055
117.646
77.324
77.000
76.685
59.771
42.008
33.723
13.881

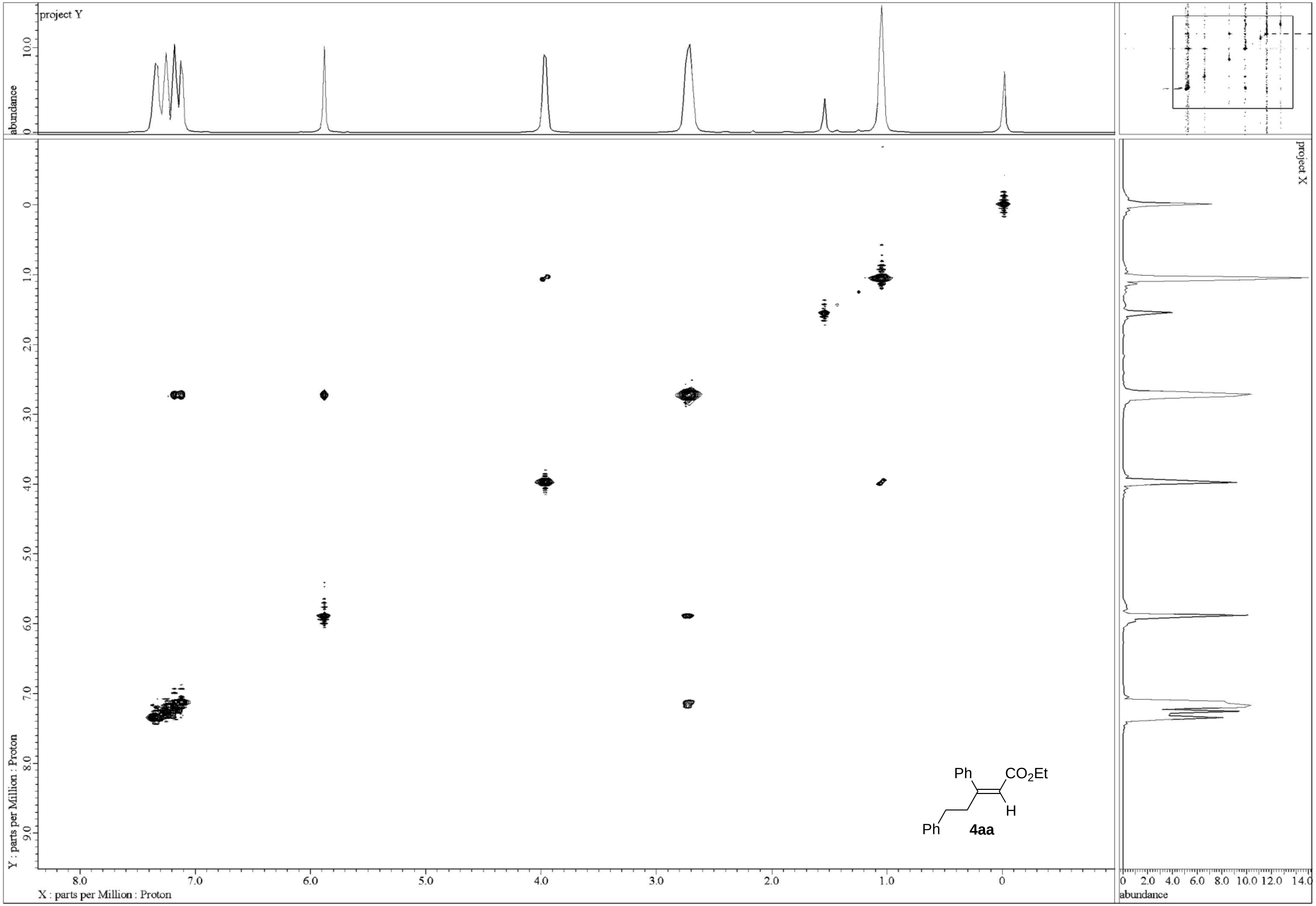
project Y
abundance
project X
Y : parts per Million : Proton
X : parts per Million : Proton
Ph
CO2Et
H
Ph
4aa

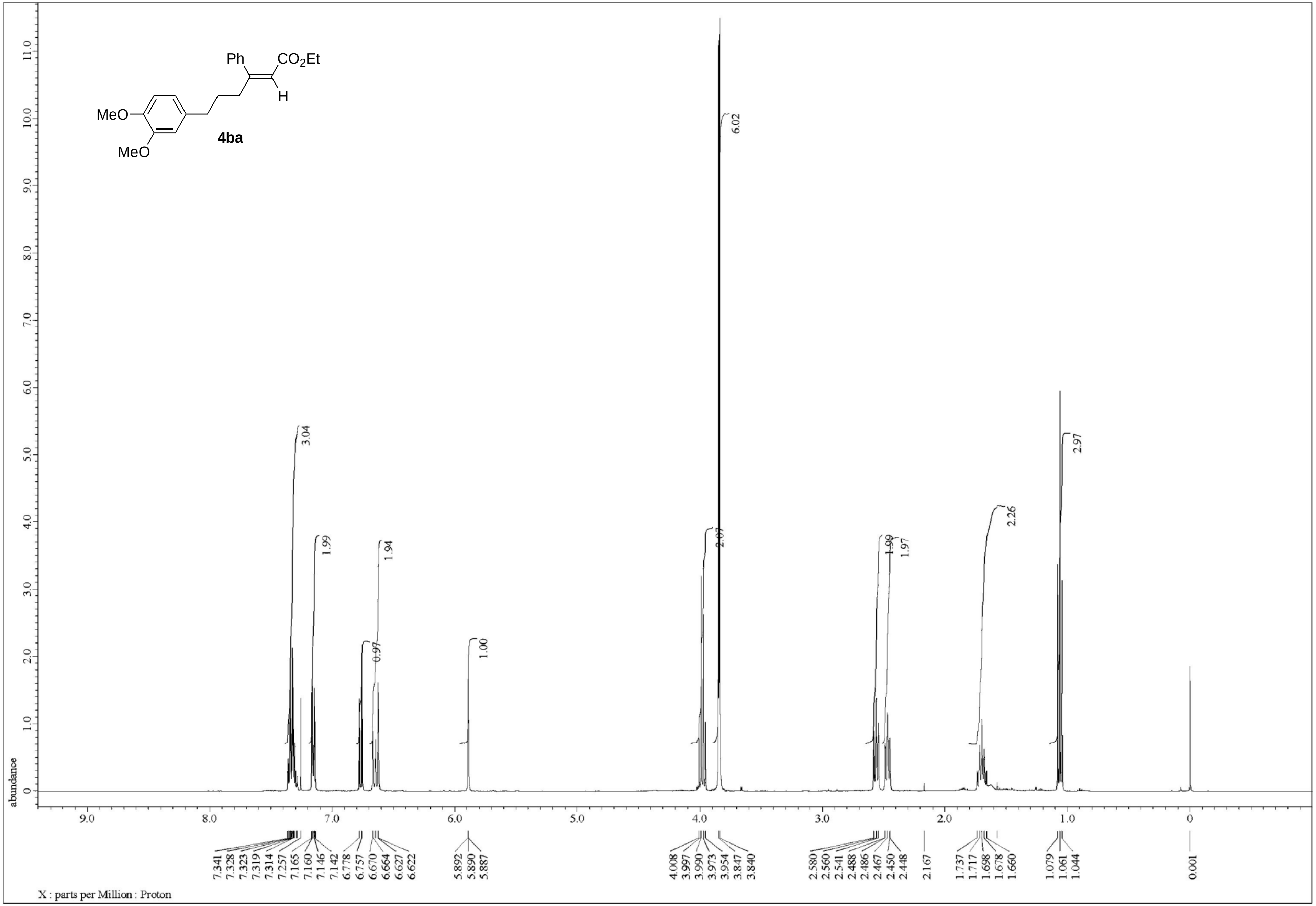
Ph
CO2Et
H
MeO
MeO
4ba
abundance
X : parts per Million : Proton

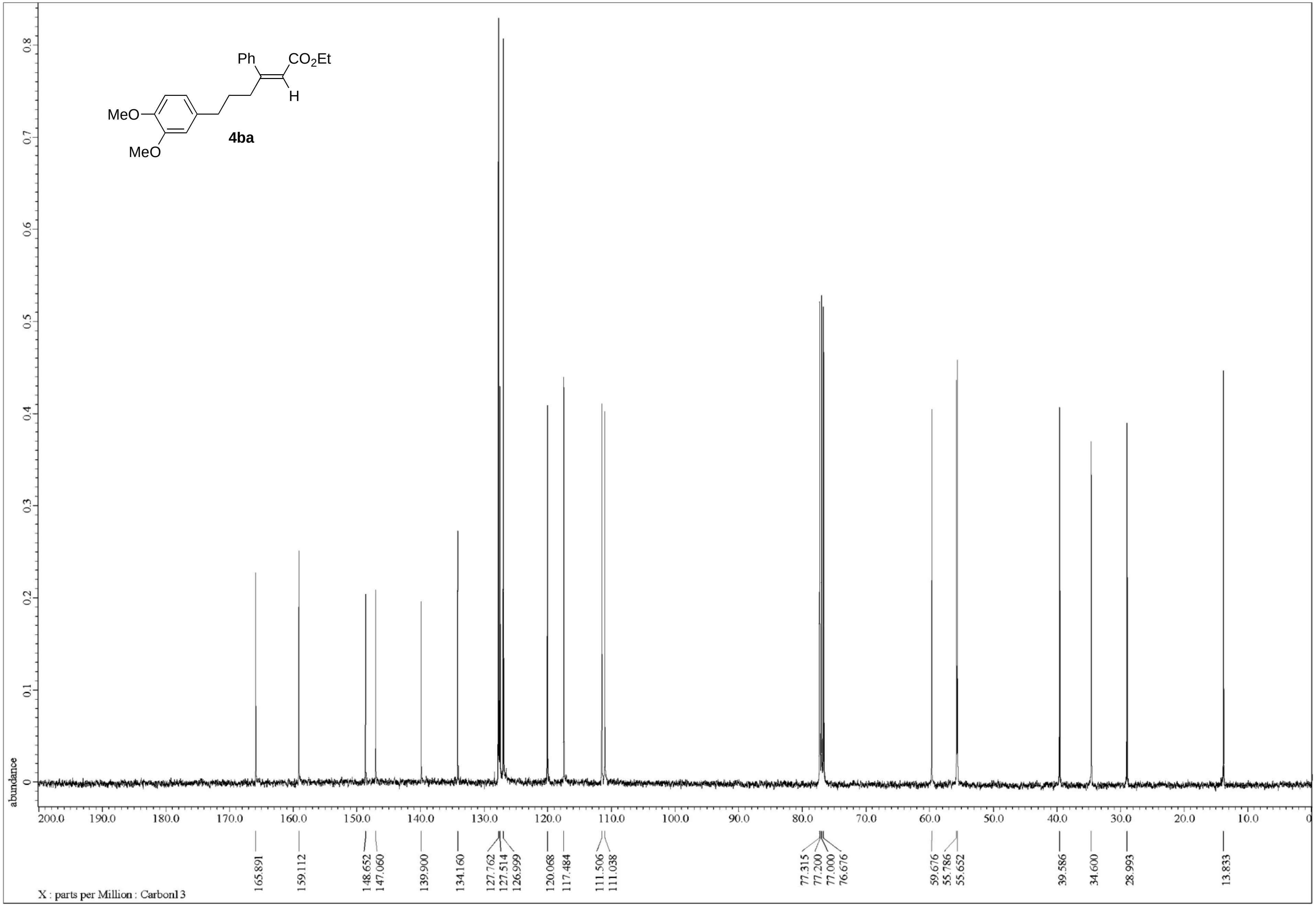

Ph
CO2Et
H
MeO
MeO
4ba
abundance
X : parts per Million : Carbon13
165.891
159.112
148.652
147.060
139.900
134.160
127.762
127.514
126.999
120.068
117.484
111.506
111.038
77.315
77.200
77.000
76.676
59.676
55.786
55.652
39.586
34.600
28.993
13.833

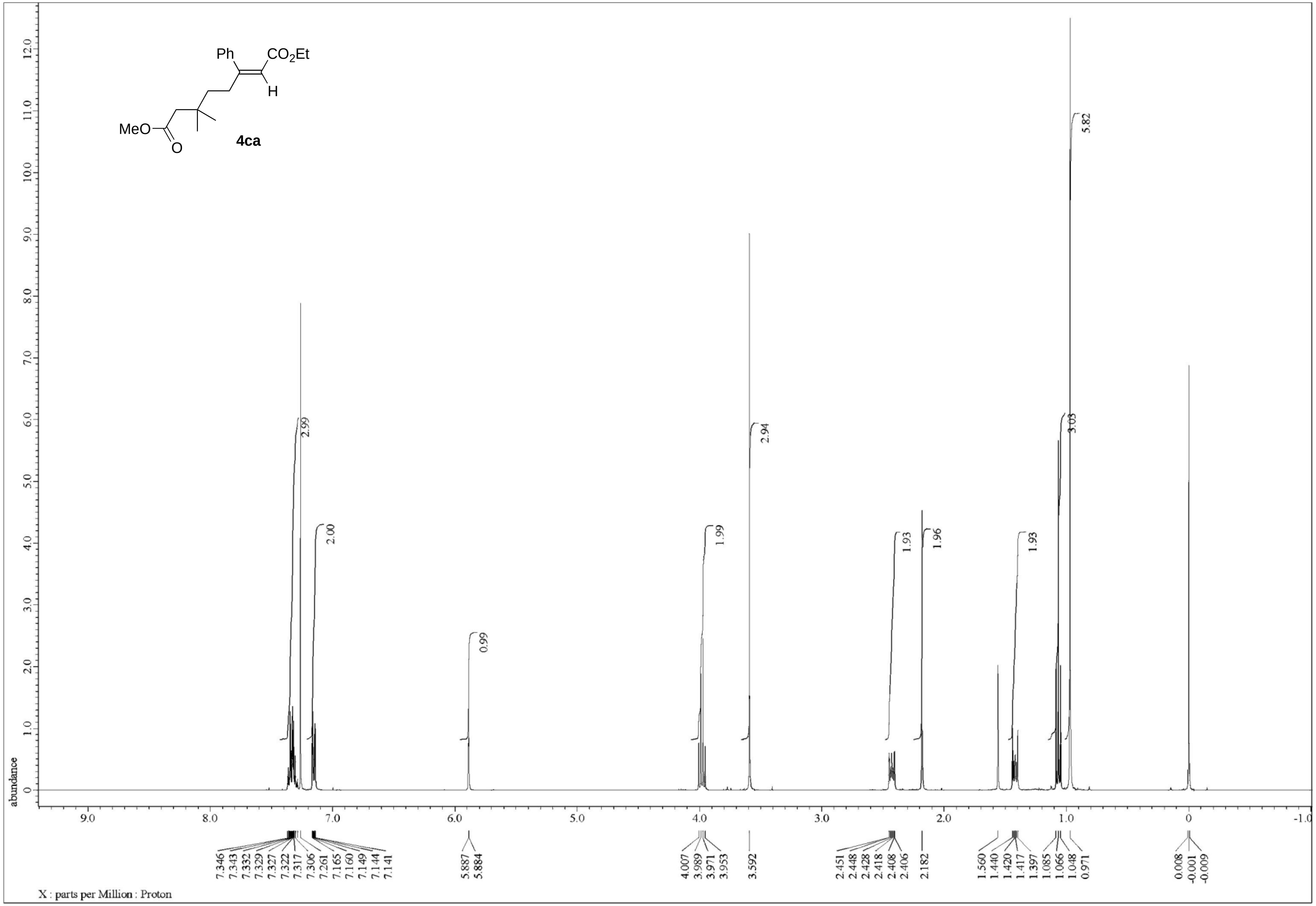
Ph
CO2Et
H
MeO
O
4ca
2.99
2.00
0.99
1.99
2.94
1.93
1.96
1.93
3.03
5.82
abundance
X : parts per Million : Proton
7.346
7.343
7.332
7.329
7.327
7.322
7.317
7.306
7.261
7.165
7.160
7.149
7.144
7.141
5.887
5.884
4.007
3.989
3.971
3.953
3.592
2.451
2.448
2.428
2.418
2.408
2.406
2.182
1.560
1.440
1.420
1.417
1.397
1.085
1.066
1.048
0.971
0.008
-0.001
-0.009

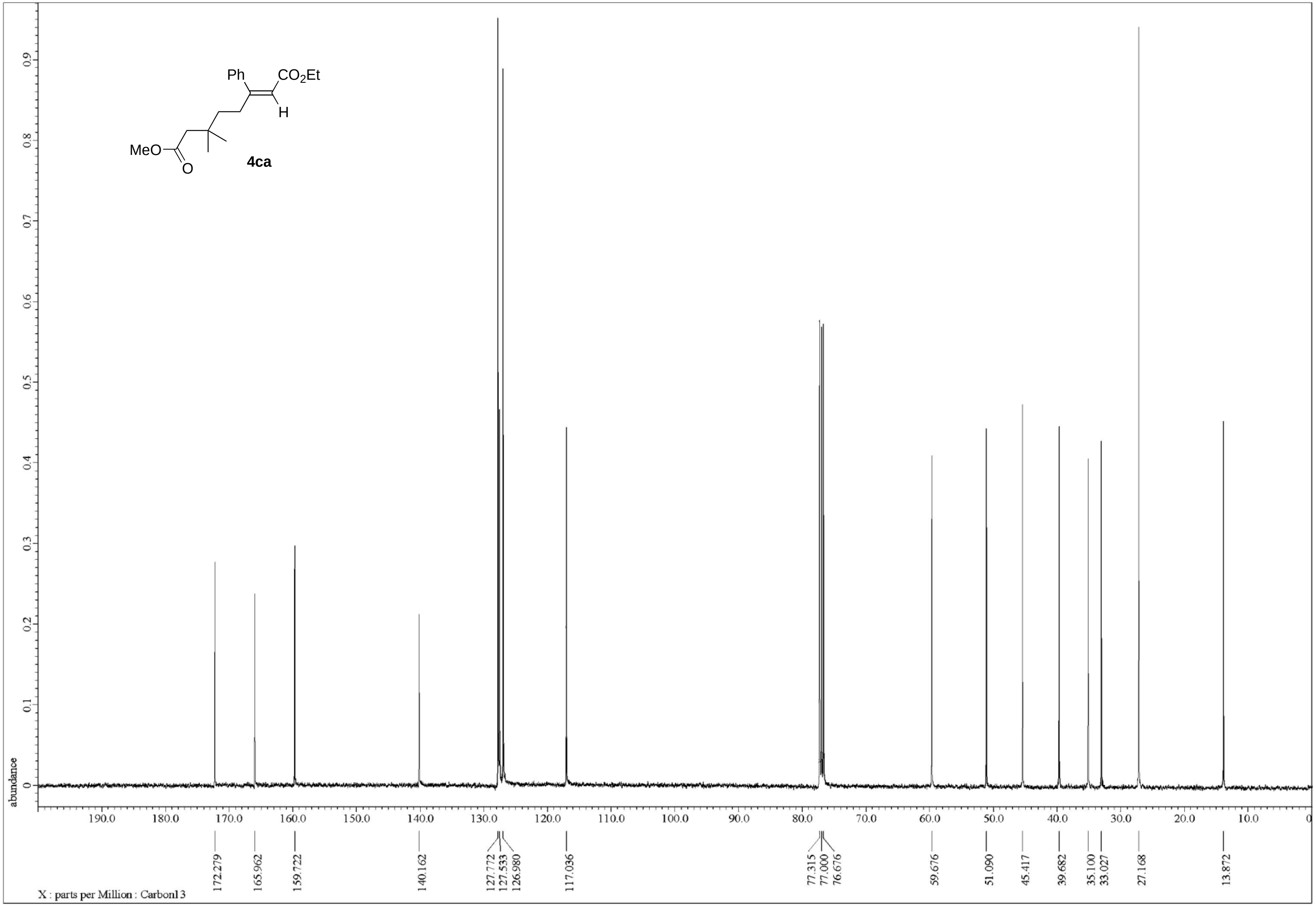

Ph
CO2Et
H
MeO
O
4ca
abundance
X : parts per Million : Carbon13
172.279
165.962
159.722
140.162
127.772
127.533
126.980
117.036
77.315
77.000
76.676
59.676
51.090
45.417
39.682
35.100
33.027
27.168
13.872

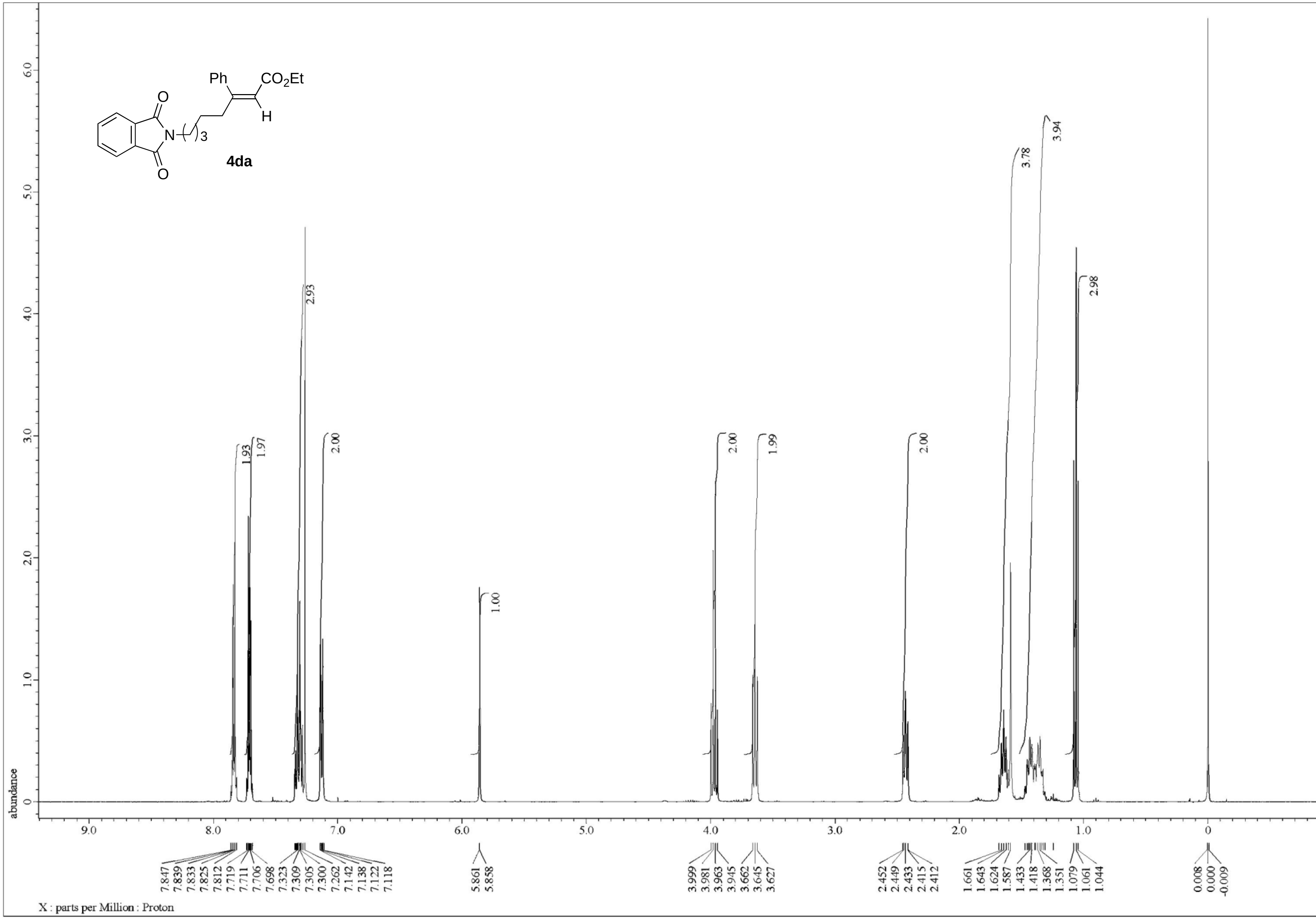
Ph
CO2Et
H
O
N
O
3
4da
abundance
X : parts per Million : Proton

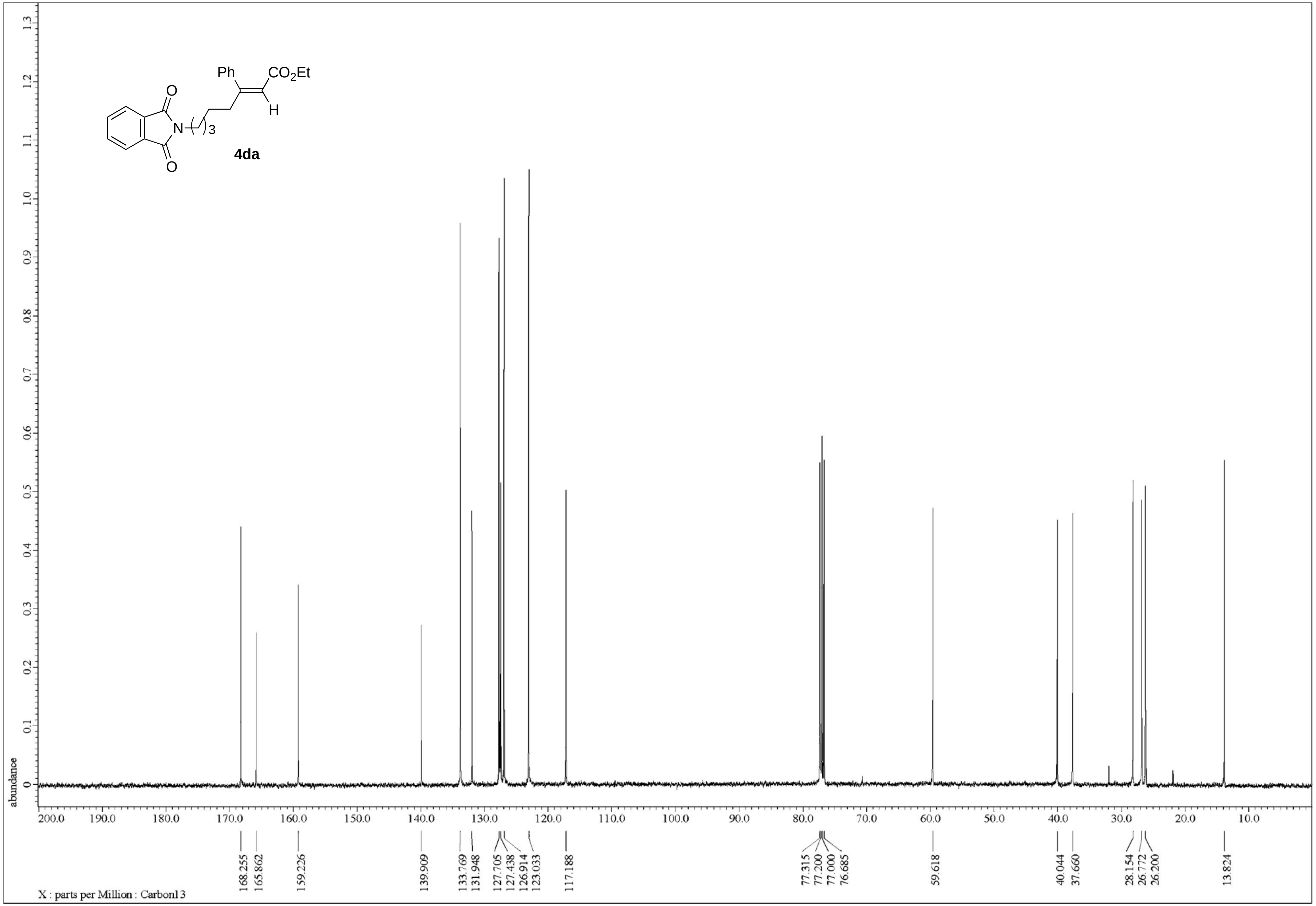
Ph
CO2Et
O
N
3
H
O
4da
abundance
X : parts per Million : Carbon13
168.255
165.862
159.226
139.909
133.769
131.948
127.705
127.438
126.914
123.033
117.188
77.315
77.200
77.000
76.685
59.618
40.044
37.660
28.154
26.772
26.200
13.824

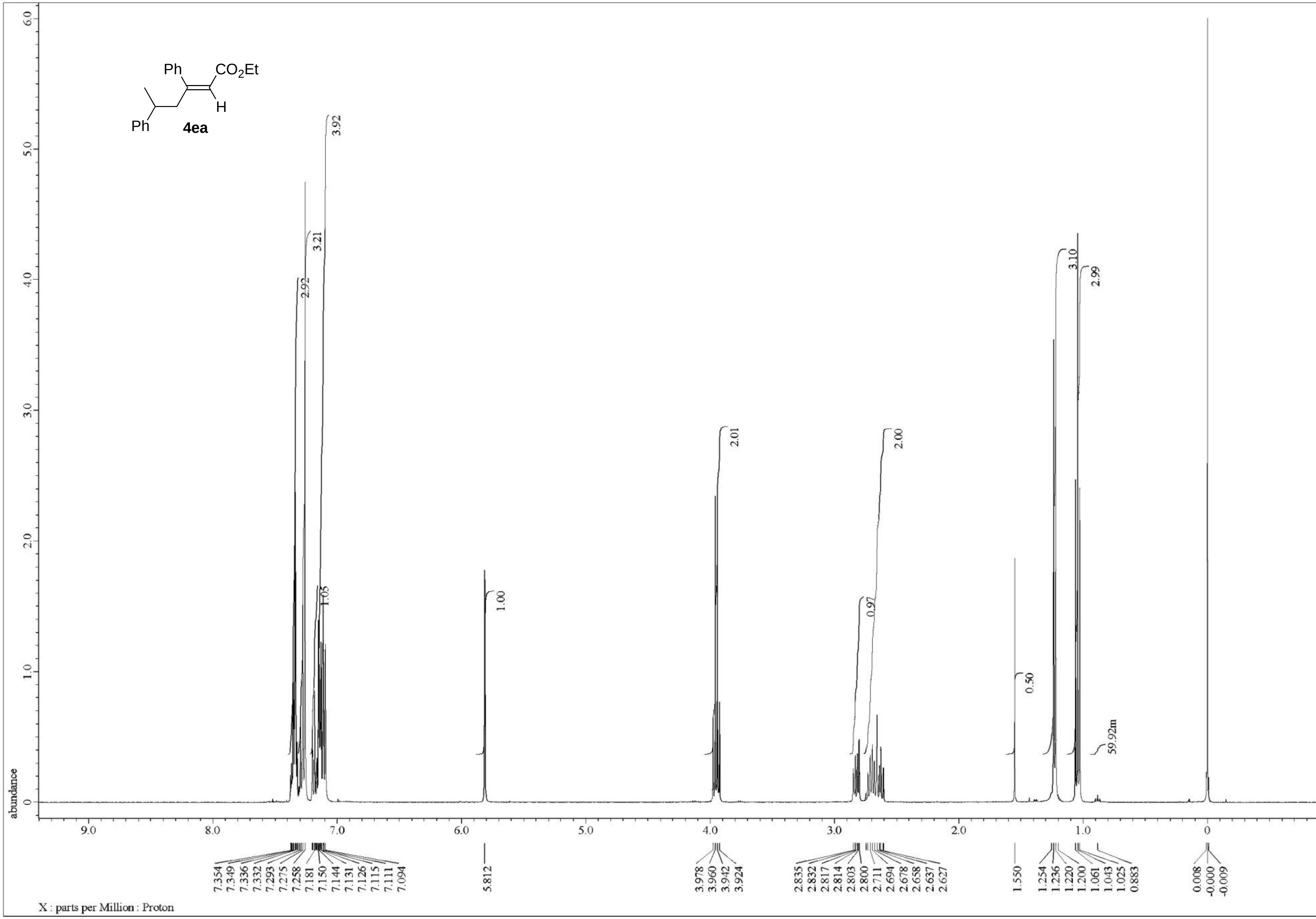
Ph
CO2Et
H
Ph
4ea
abundance
X : parts per Million : Proton

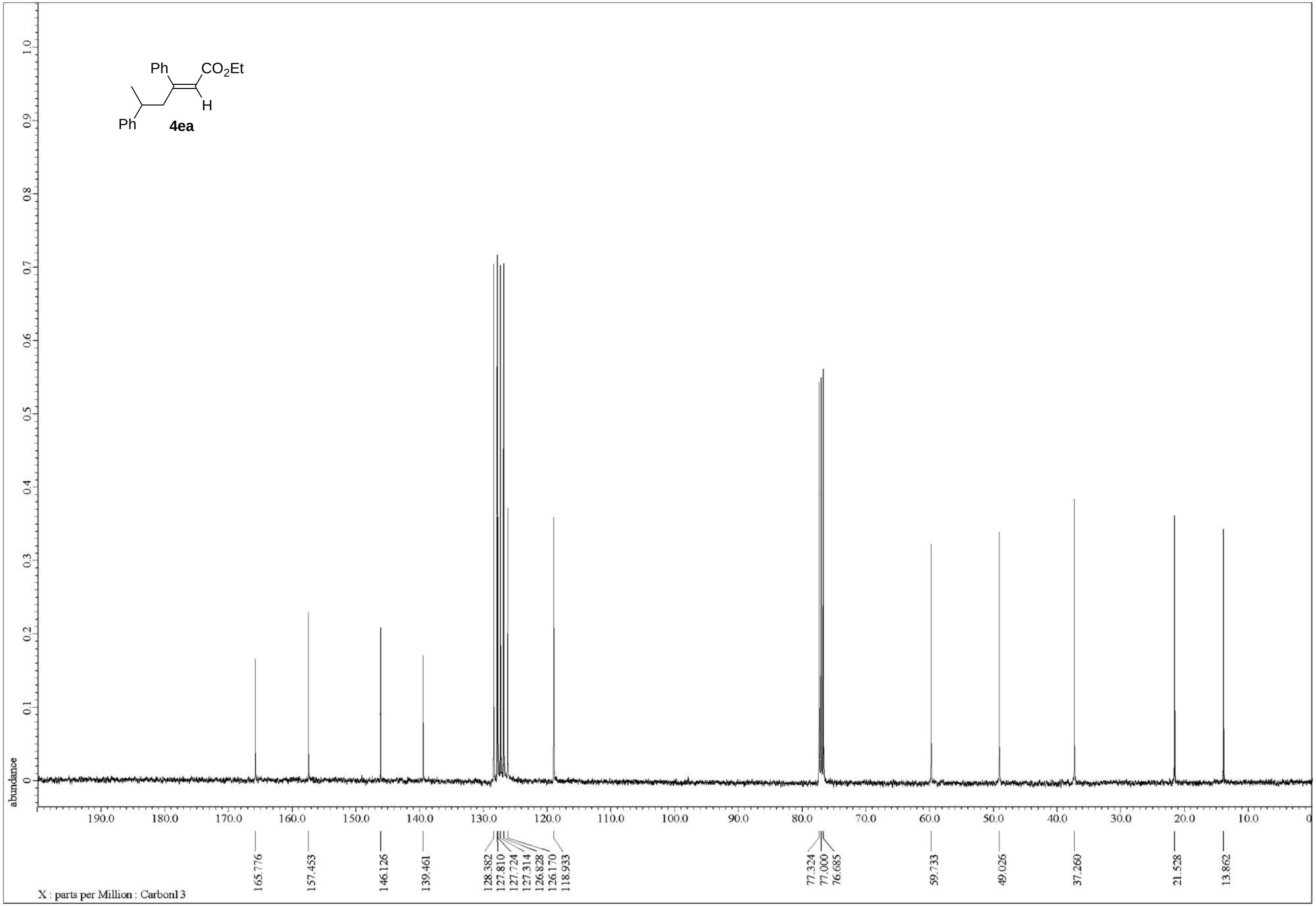

Ph
CO2Et
H
Ph
4ea
abundance
X : parts per Million : Carbon13
165.776
157.453
146.126
139.461
128.382
127.810
127.724
127.314
126.828
126.170
118.933
77.324
77.000
76.685
59.733
49.026
37.260
21.528
13.862

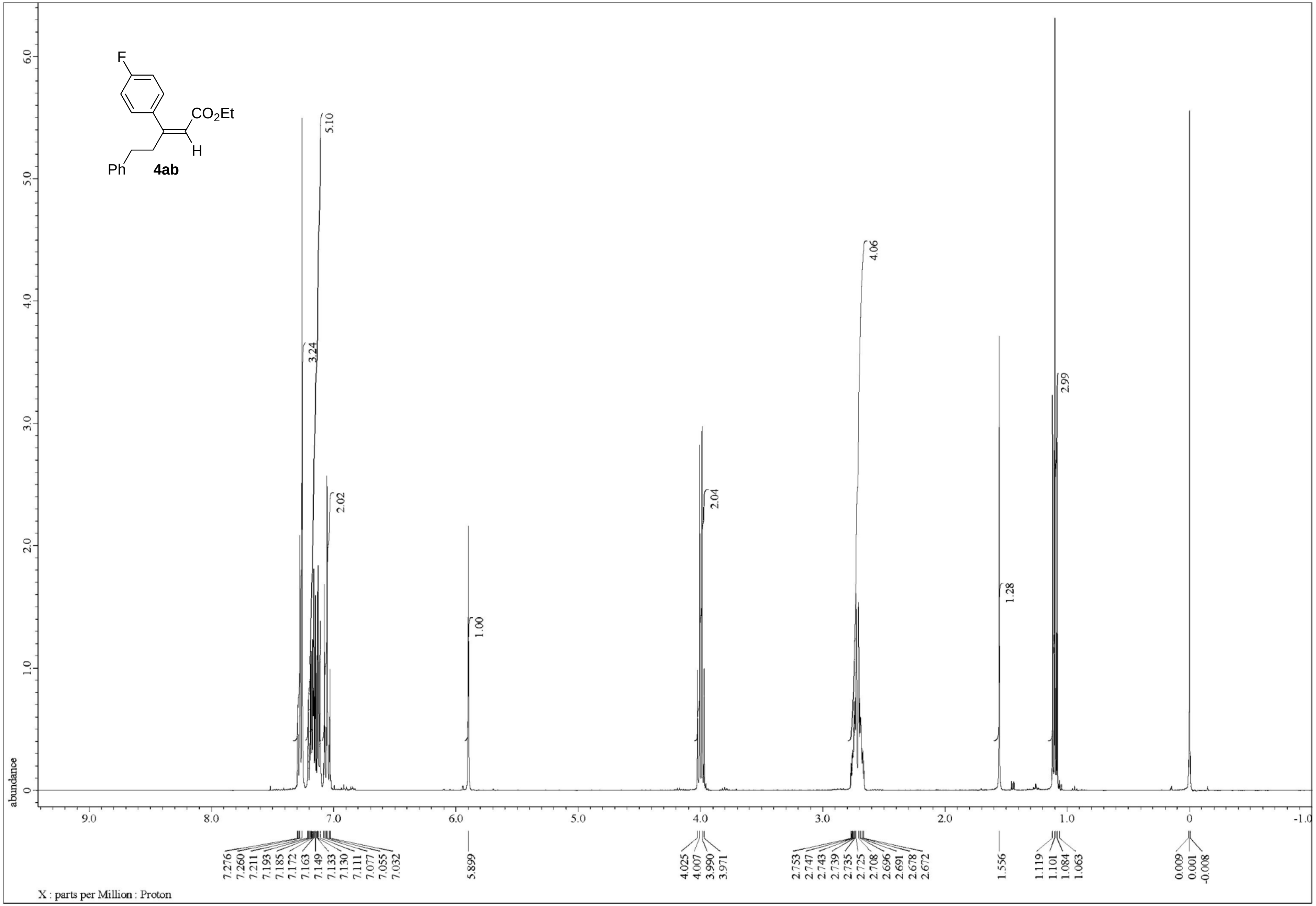
F
CO2Et
H
Ph
4ab
abundance
X : parts per Million : Proton
5.10
3.24
2.02
1.00
2.04
4.06
1.28
2.99
7.276
7.260
7.211
7.193
7.185
7.172
7.163
7.149
7.133
7.130
7.111
7.077
7.055
7.032
5.899
4.025
4.007
3.990
3.971
2.753
2.747
2.743
2.739
2.735
2.725
2.708
2.696
2.691
2.678
2.672
1.556
1.119
1.101
1.084
1.063
0.009
0.001
-0.008

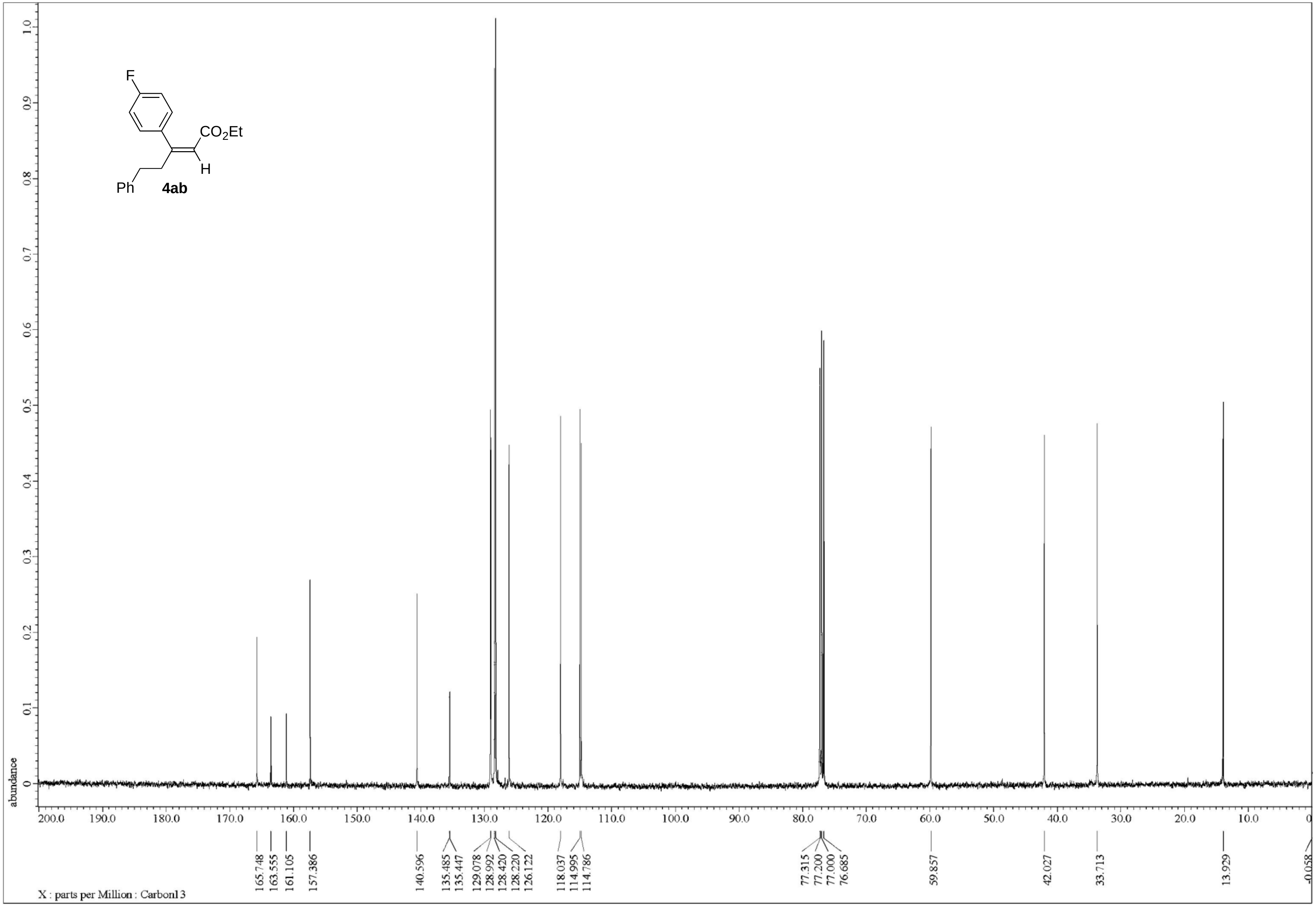
F
CO2Et
H
Ph
4ab
abundance
X : parts per Million : Carbon13
165.748
163.555
161.105
157.386
140.596
135.485
135.447
129.078
128.992
128.420
128.220
126.122
118.037
114.995
114.786
77.315
77.200
77.000
76.685
59.857
42.027
33.713
13.929
-0.058

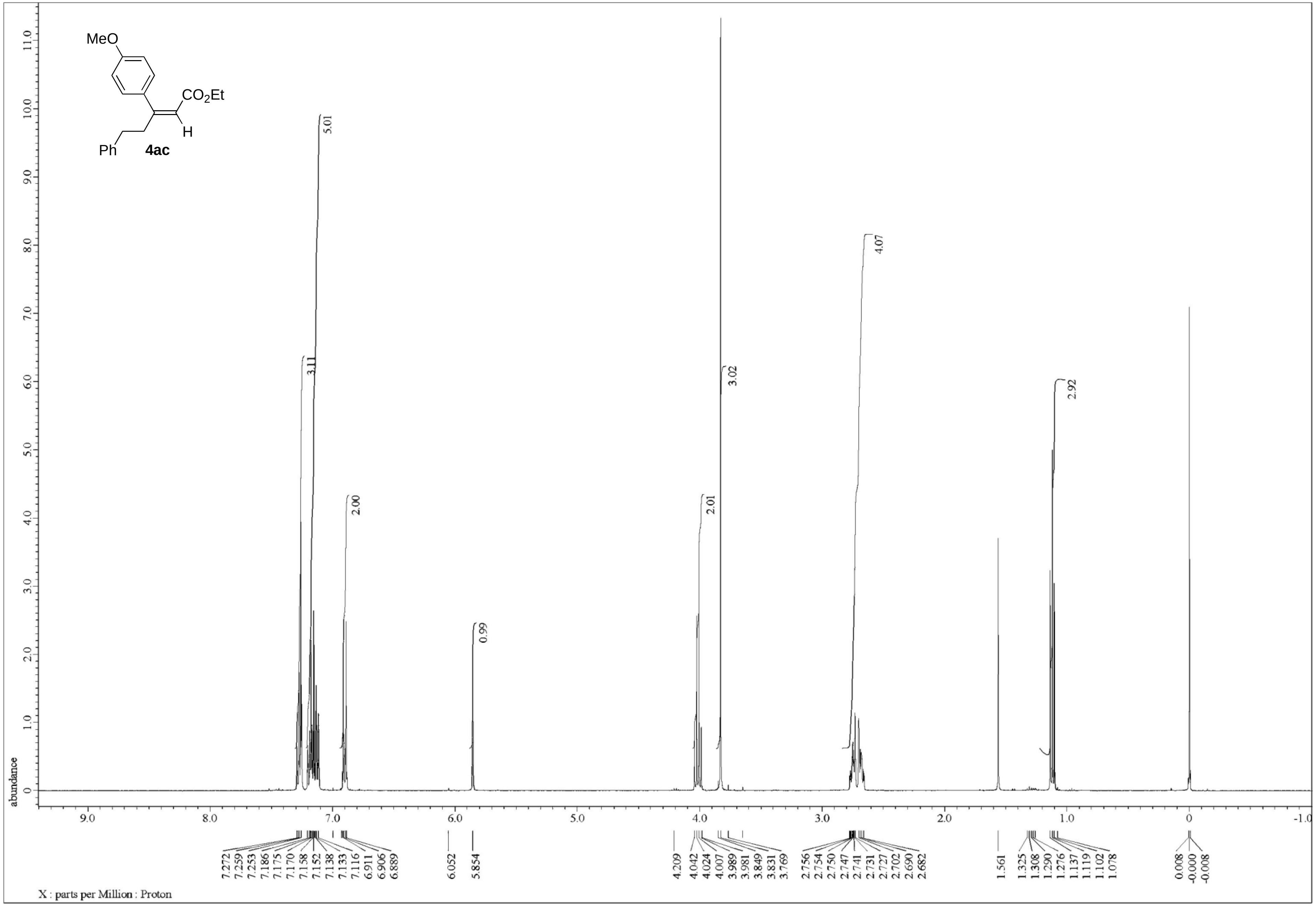
MeO
CO2Et
H
Ph
4ac
abundance
X : parts per Million : Proton
5.01
3.11
2.00
0.99
2.01
3.02
4.07
2.92

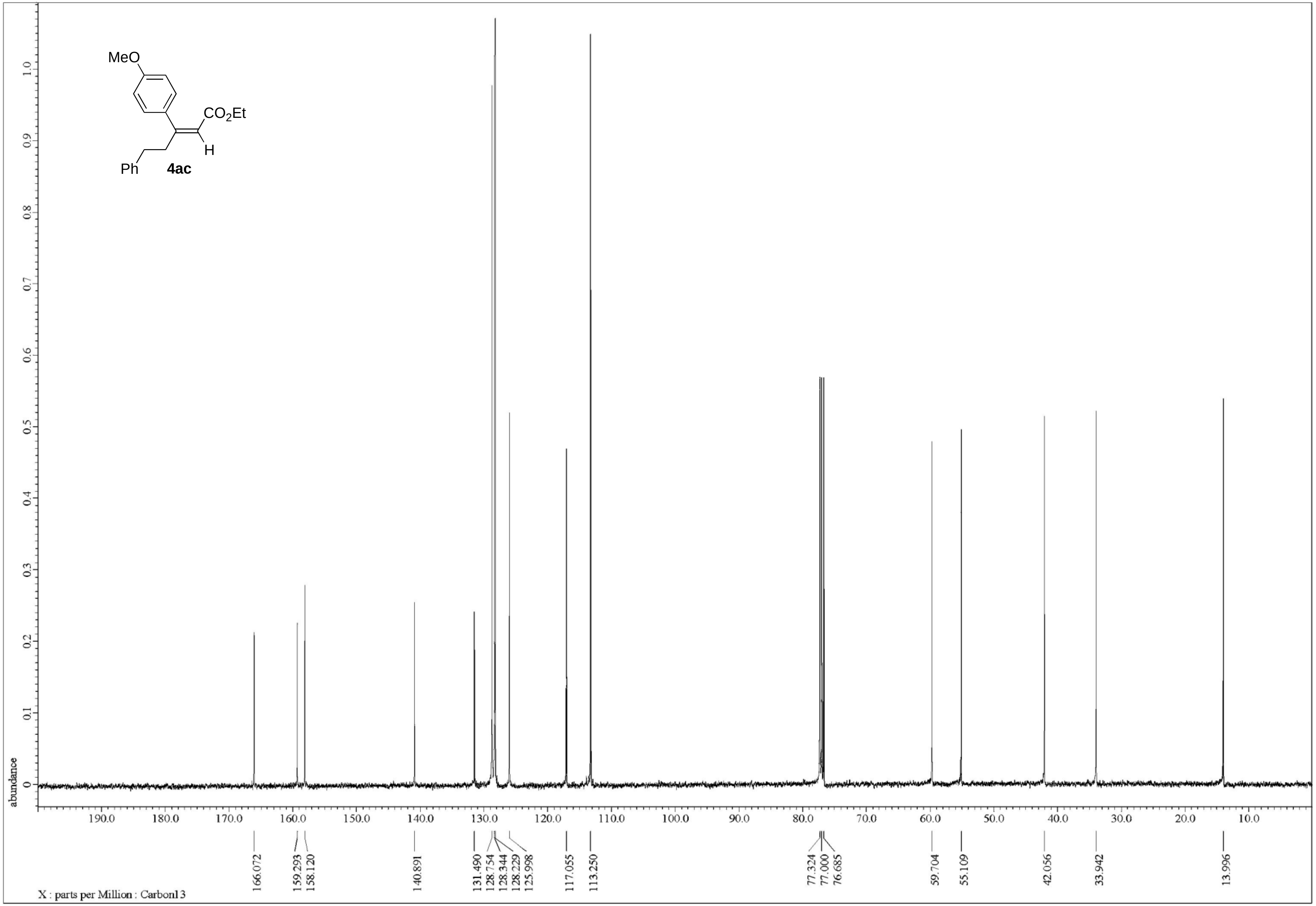
MeO
CO2Et
H
Ph
4ac
abundance
190.0 180.0 170.0 160.0 150.0 140.0 130.0 120.0 110.0 100.0 90.0 80.0 70.0 60.0 50.0 40.0 30.0 20.0 10.0
166.072 159.293 158.120 140.891 131.490 128.754 128.344 128.229 125.998 117.055 113.250 77.324 77.000 76.685 59.704 55.109 42.056 33.942 13.996
X : parts per Million : Carbon13

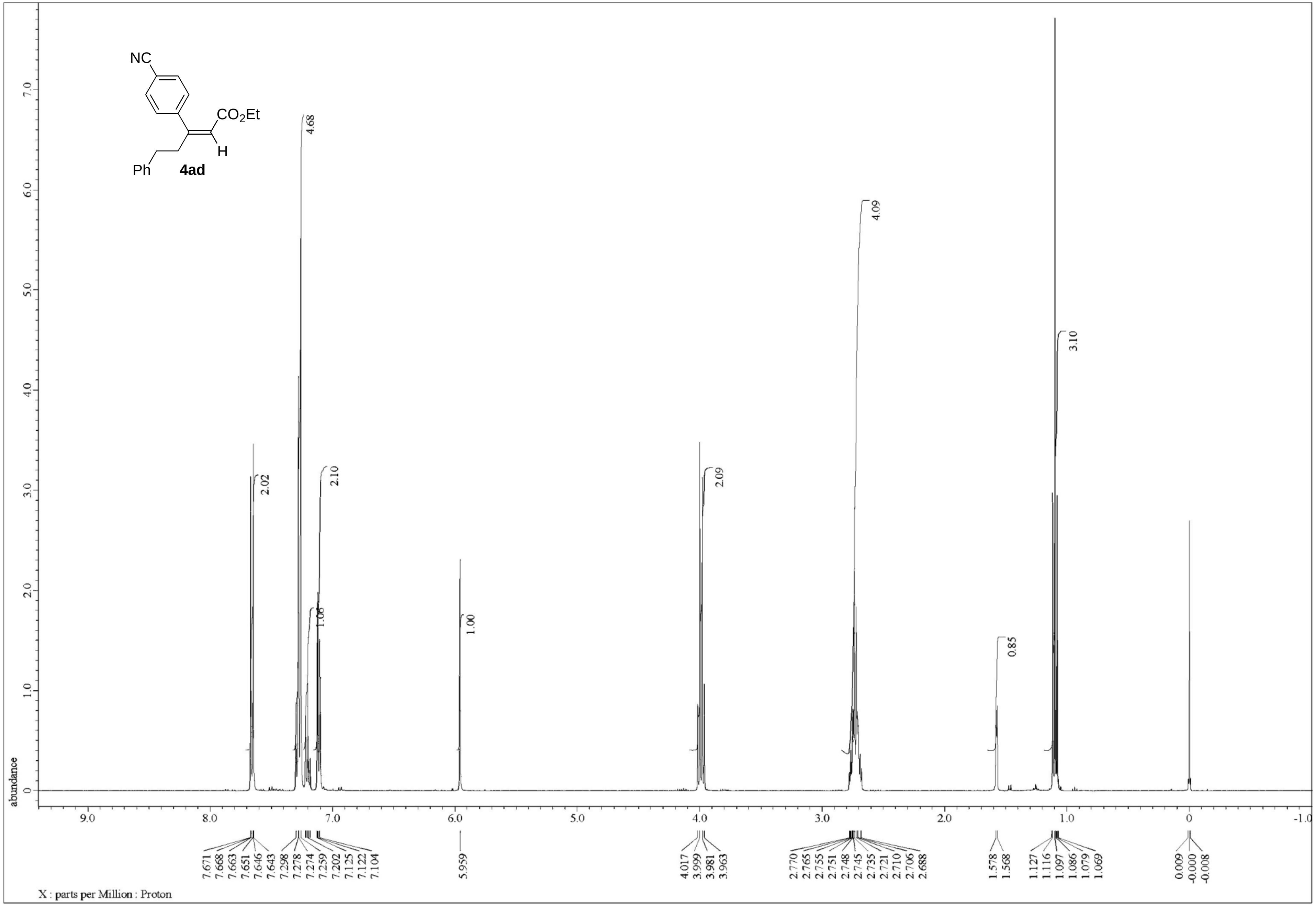
NC
CO2Et
H
Ph
4ad
abundance
X : parts per Million : Proton
4.68
2.02
2.10
1.06
1.00
2.09
4.09
0.85
3.10
7.671
7.668
7.663
7.651
7.646
7.643
7.298
7.278
7.274
7.259
7.202
7.125
7.122
7.104
5.959
4.017
3.999
3.981
3.963
2.770
2.765
2.755
2.751
2.748
2.745
2.735
2.721
2.710
2.706
2.688
1.578
1.568
1.127
1.116
1.097
1.086
1.079
1.069
0.009
-0.000
-0.008

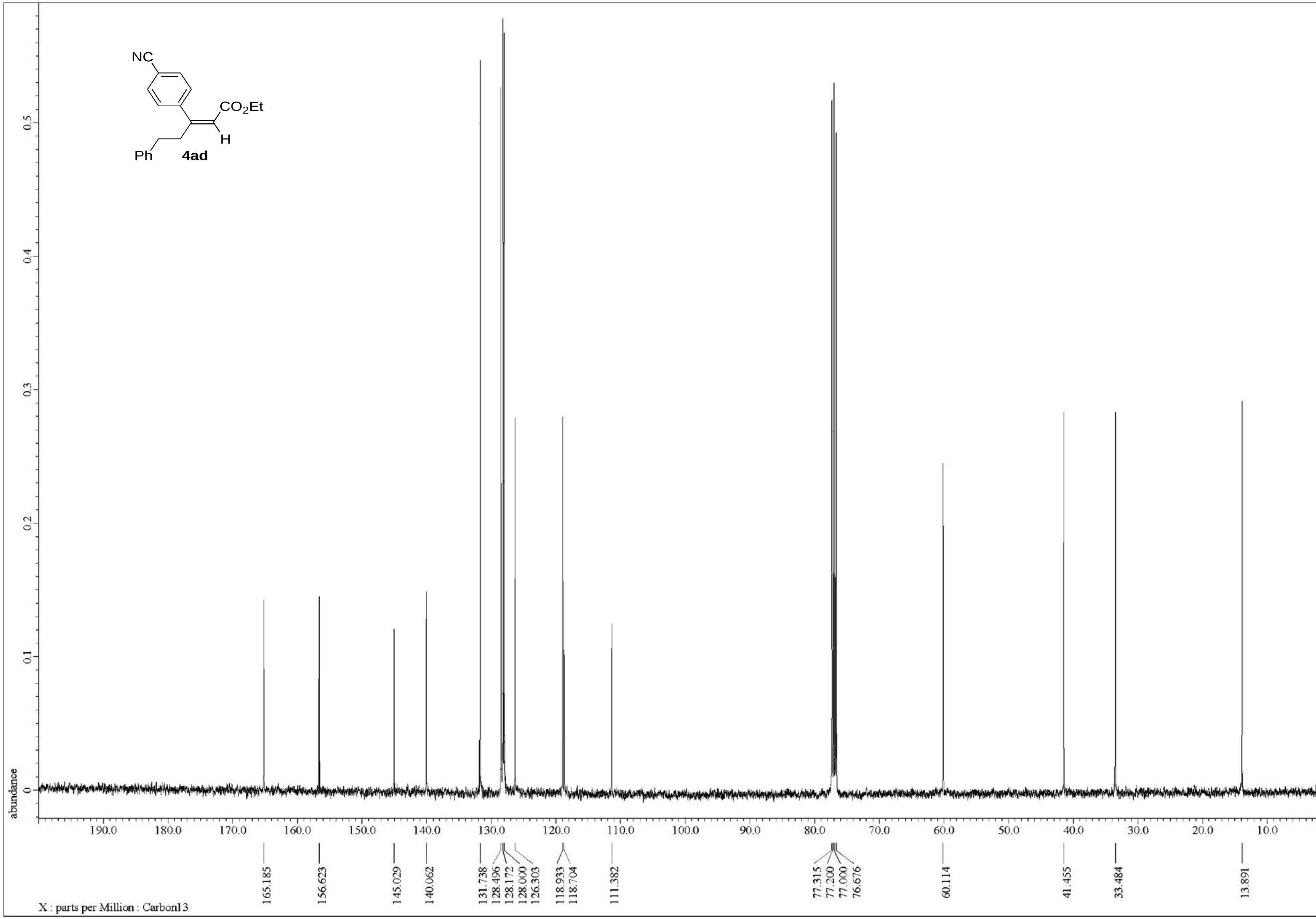
NC
CO2Et
H
Ph
4ad
abundance
X : parts per Million : Carbon13
165.185
156.623
145.029
140.062
131.738
128.496
128.172
128.000
126.303
118.933
118.704
111.382
77.315
77.200
77.000
76.676
60.114
41.455
33.484
13.891

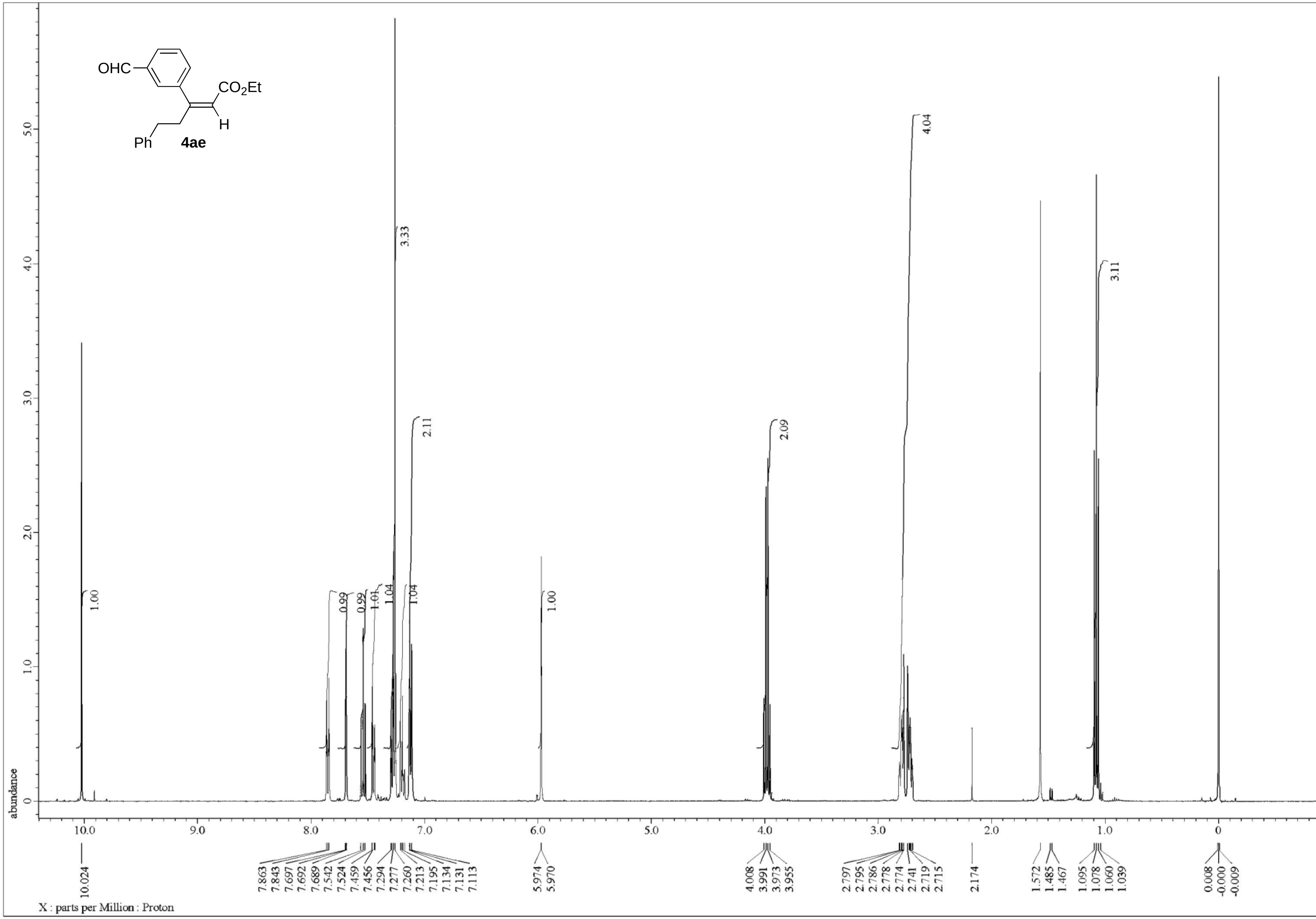
OHC
CO2Et
H
Ph
4ae
abundance
X : parts per Million : Proton
1.00
0.99
0.92
1.01
1.04
3.33
1.04
2.11
1.00
2.09
4.04
3.11
10.024
7.863
7.843
7.697
7.692
7.689
7.542
7.524
7.459
7.456
7.294
7.277
7.260
7.213
7.195
7.134
7.131
7.113
5.974
5.970
4.008
3.991
3.973
3.955
2.797
2.795
2.786
2.778
2.774
2.741
2.719
2.715
2.174
1.572
1.485
1.467
1.095
1.078
1.060
1.039
0.008
-0.000
-0.009

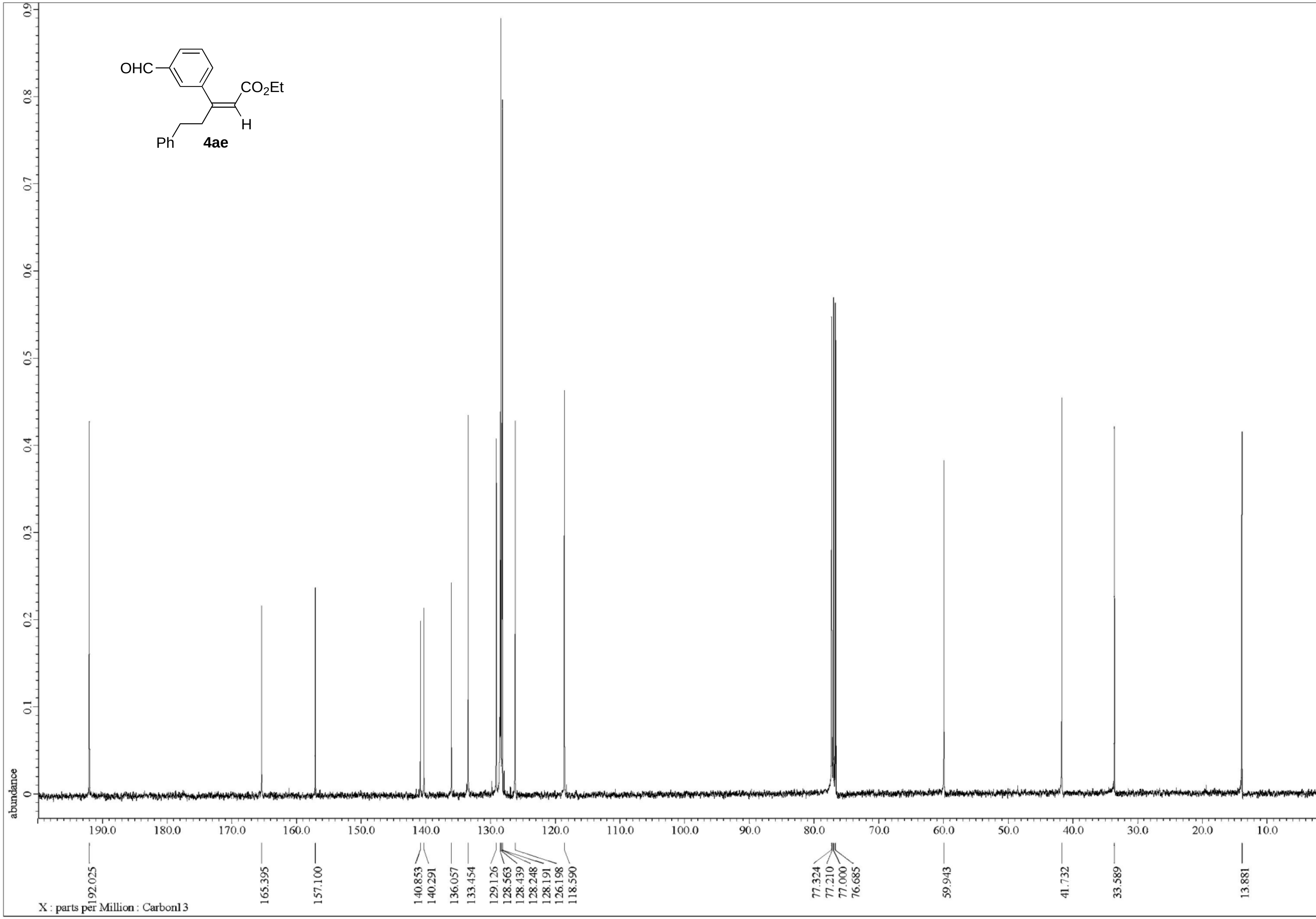
OHC
CO2Et
H
Ph
4ae
abundance
X : parts per Million : Carbon13
192.025
165.395
157.100
140.853
140.291
136.057
133.454
129.126
128.563
128.439
128.248
128.191
126.198
118.590
77.324
77.210
77.000
76.685
59.943
41.732
33.589
13.881

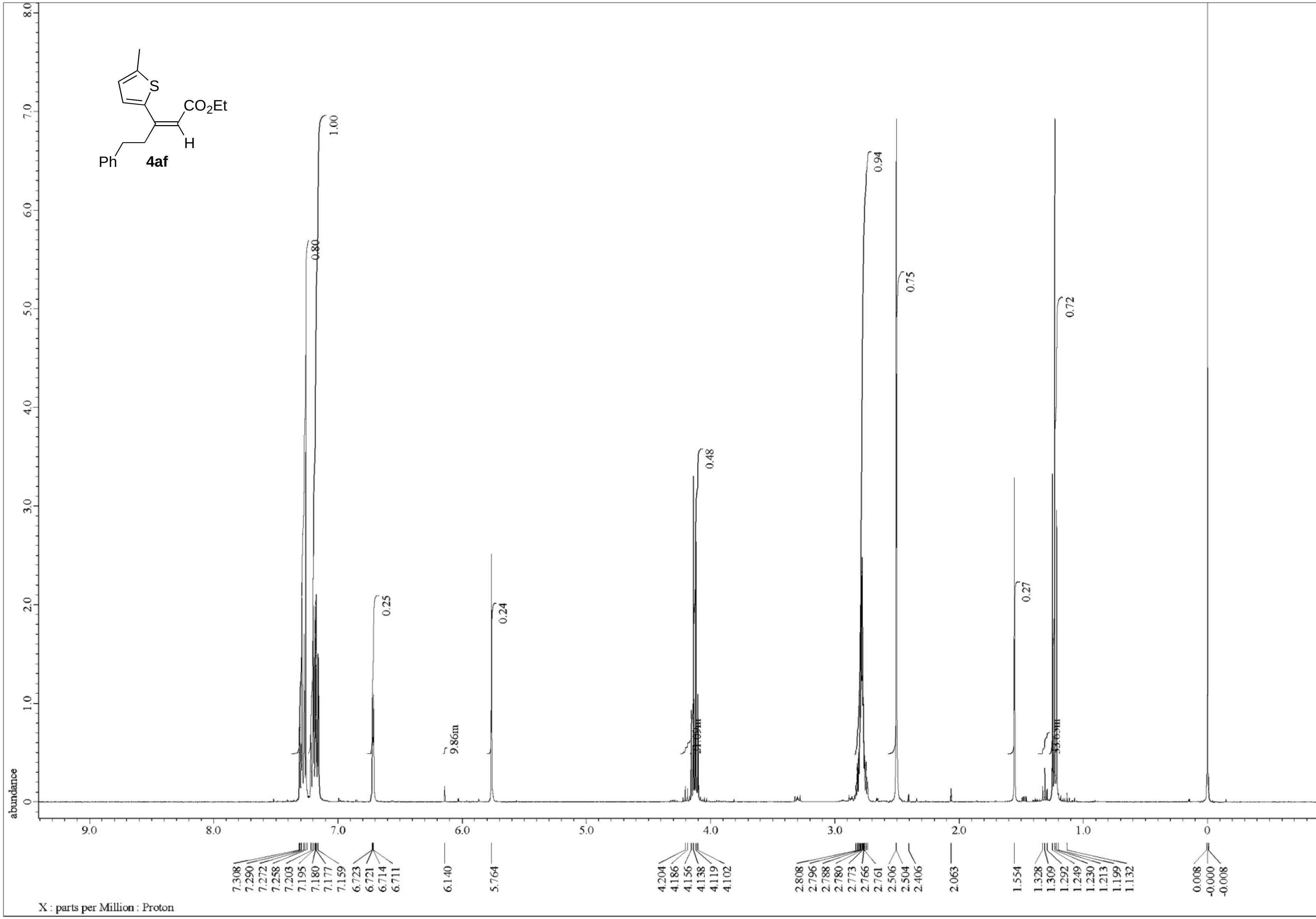

CO2Et
S
H
Ph
4af
abundance
X : parts per Million : Proton

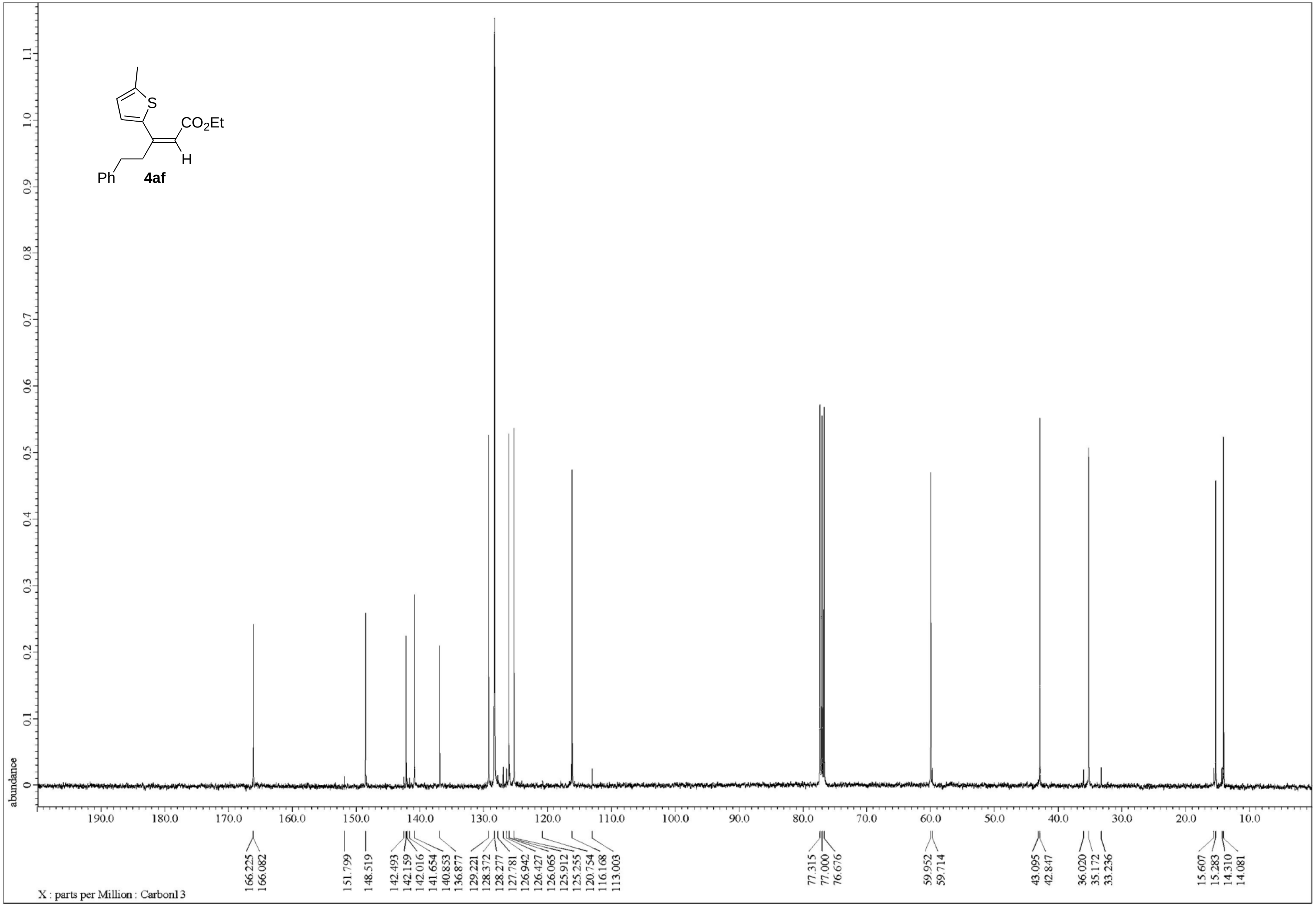

CO2Et
S
H
Ph
4af
abundance
X : parts per Million : Carbon13
166.225
166.082
151.799
148.519
142.493
142.159
142.016
141.654
140.853
136.877
129.221
128.372
128.277
127.781
126.942
126.427
126.065
125.912
125.255
120.754
116.168
113.003
77.315
77.000
76.676
59.952
59.714
43.095
42.847
36.020
35.172
33.236
15.607
15.283
14.310
14.081

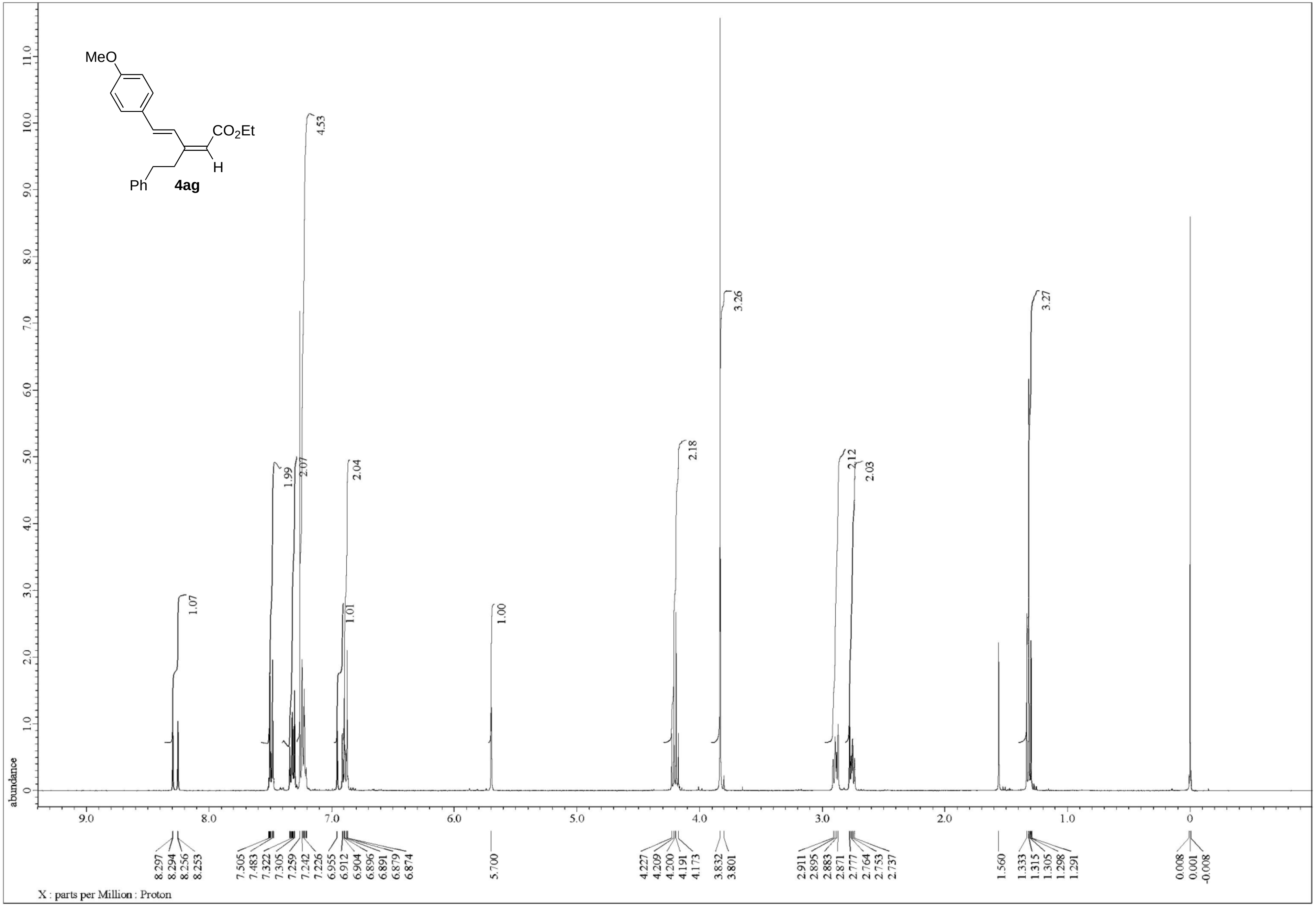
MeO
CO_2Et
H
Ph
4ag
abundance
X : parts per Million : Proton
1.07
1.99
2.07
4.53
2.04
1.01
1.00
2.18
3.26
2.12
2.03
3.27
8.297
8.294
8.256
8.253
7.505
7.483
7.322
7.305
7.259
7.242
7.226
6.955
6.912
6.904
6.896
6.891
6.879
6.874
5.700
4.227
4.209
4.200
4.191
4.173
3.832
3.801
2.911
2.895
2.883
2.871
2.777
2.764
2.753
2.737
1.560
1.333
1.315
1.305
1.298
1.291
0.008
0.001
-0.008

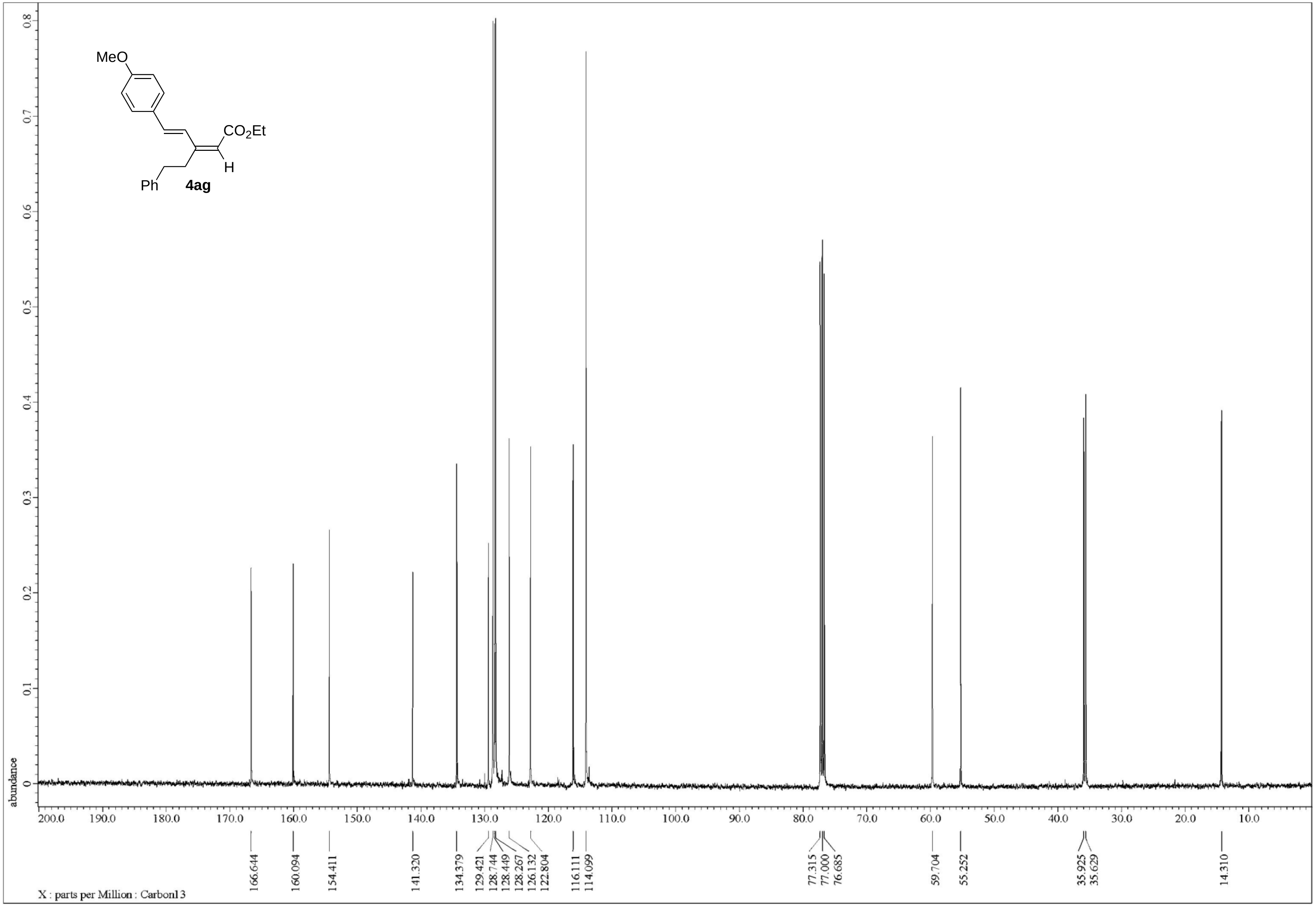

MeO
CO2Et
H
Ph
4ag
abundance
X : parts per Million : Carbon13
166.644
160.094
154.411
141.320
134.379
129.421
128.744
128.449
128.267
126.132
122.804
116.111
114.099
77.315
77.000
76.685
59.704
55.252
35.925
35.629
14.310

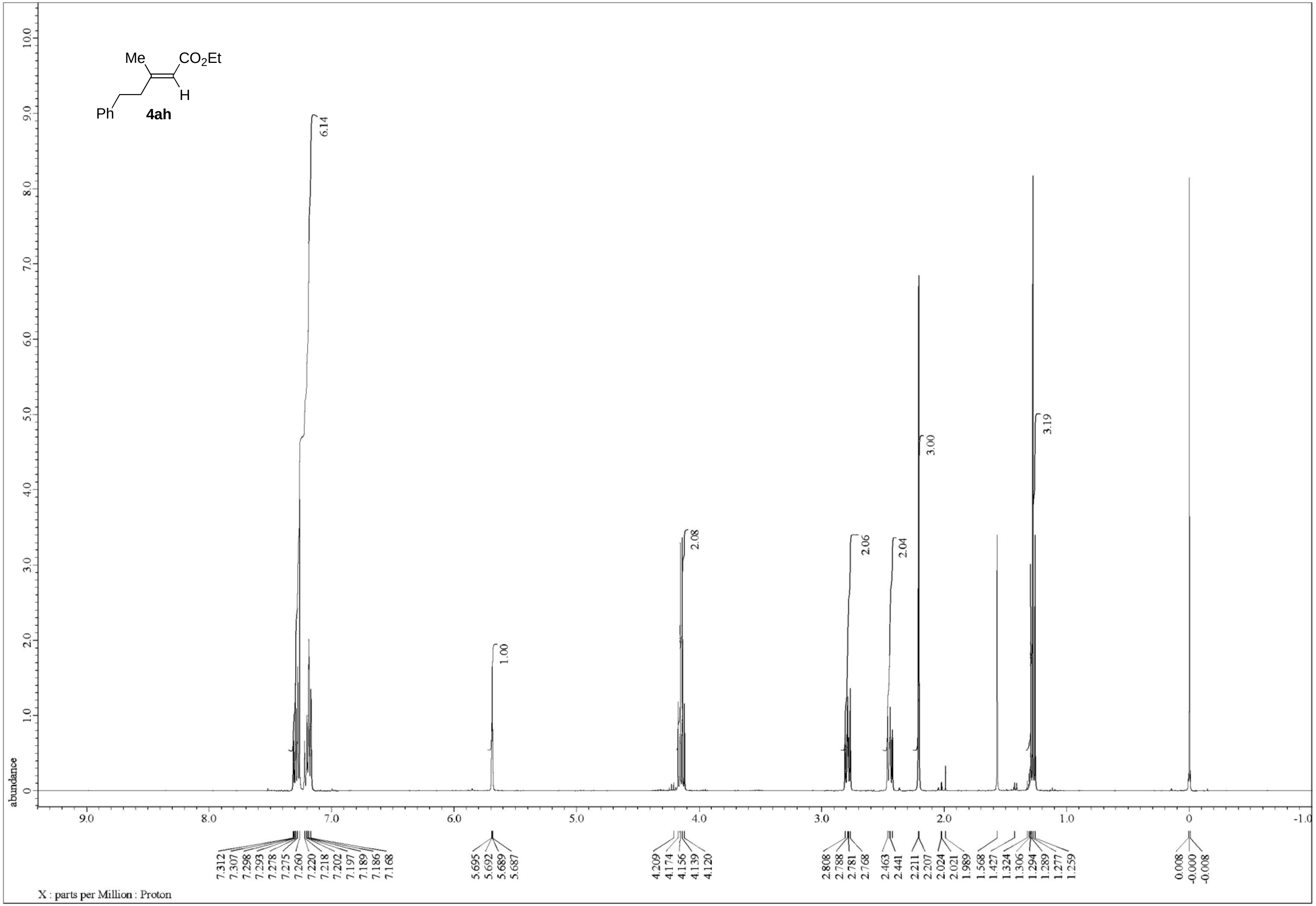
Me
CO2Et
H
Ph
4ah
abundance
X : parts per Million : Proton

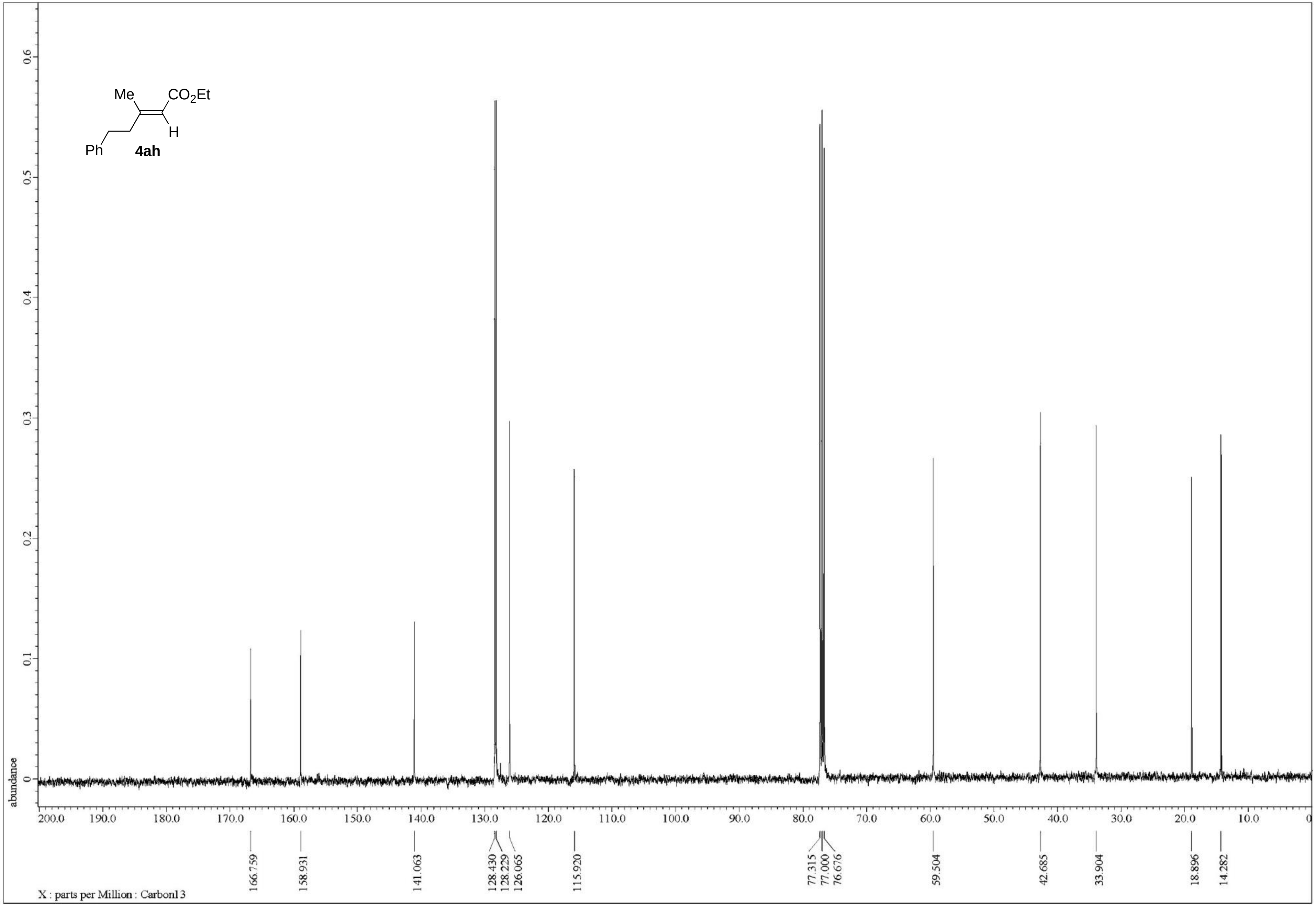
Me
CO2Et
H
Ph
4ah
abundance
X : parts per Million : Carbon13
166.759
158.931
141.063
128.430
128.229
126.065
115.920
77.315
77.000
76.676
59.504
42.685
33.904
18.896
14.282

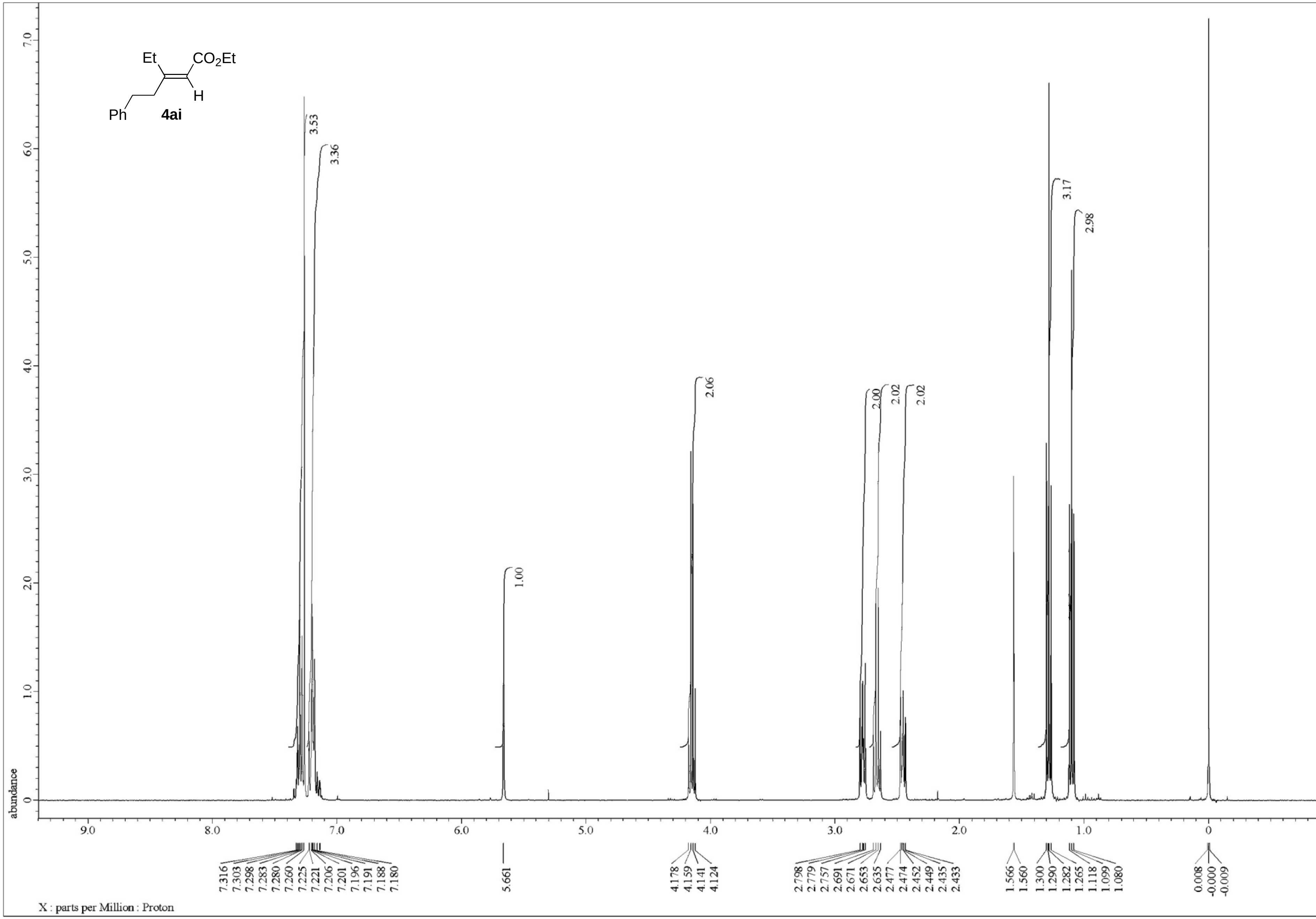
Et
CO_2Et
H
Ph
4ai
3.53
3.36
1.00
2.06
2.00
2.02
2.02
3.17
2.98
abundance
X : parts per Million : Proton
7.316
7.303
7.298
7.283
7.280
7.260
7.225
7.221
7.206
7.201
7.196
7.191
7.188
7.180
5.661
4.178
4.159
4.141
4.124
2.798
2.779
2.757
2.691
2.671
2.653
2.635
2.477
2.474
2.452
2.449
2.435
2.433
1.566
1.560
1.300
1.290
1.282
1.265
1.118
1.099
1.080
0.008
-0.000
-0.009

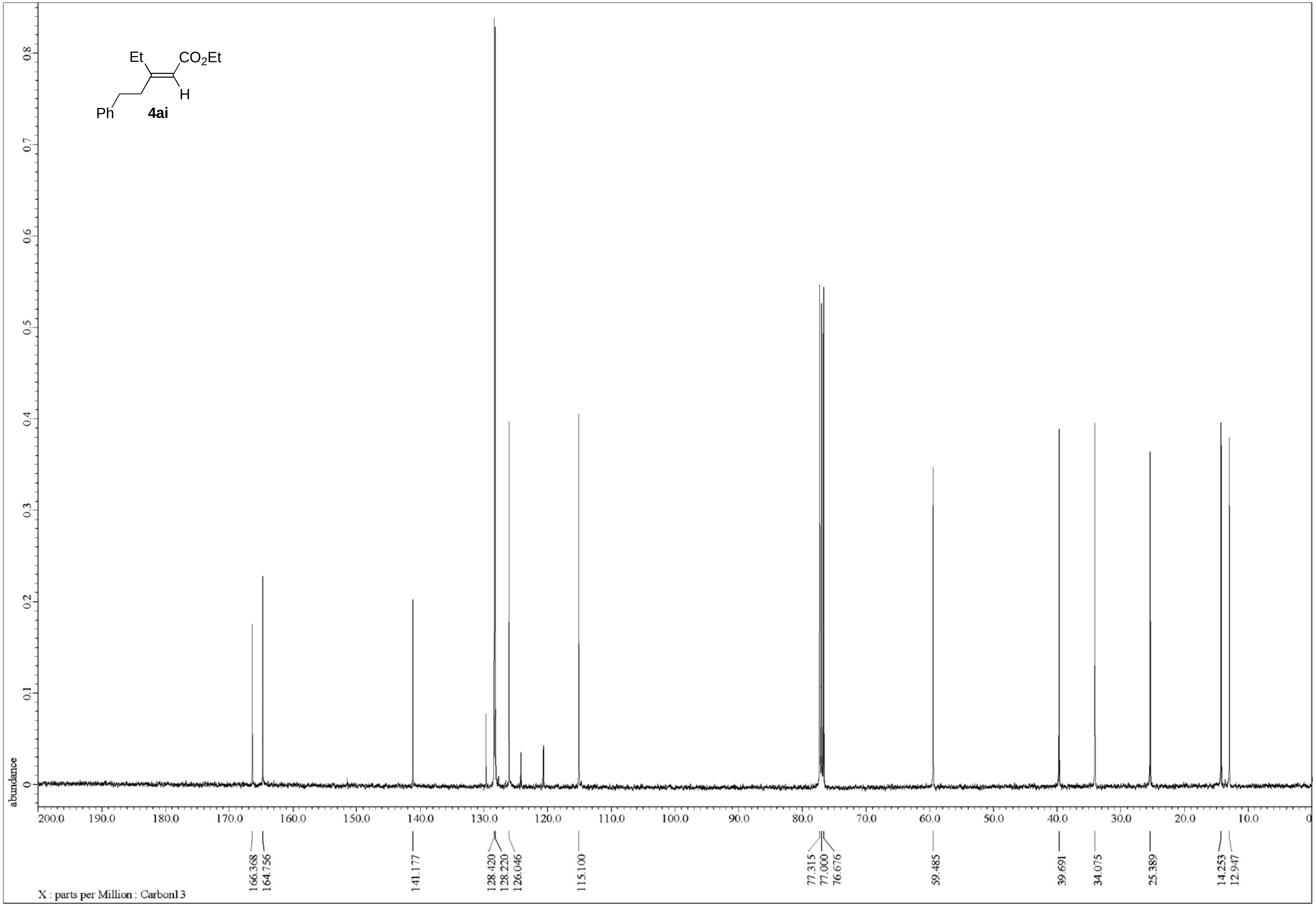

Et
CO2Et
H
Ph
4ai
abundance
0.8
0.7
0.6
0.5
0.4
0.3
0.2
0.1
0
200.0 190.0 180.0 170.0 160.0 150.0 140.0 130.0 120.0 110.0 100.0 90.0 80.0 70.0 60.0 50.0 40.0 30.0 20.0 10.0 0
166.368
164.756
141.177
128.420
128.220
126.046
115.100
77.315
77.000
76.676
59.485
39.691
34.075
25.389
14.253
12.947
X : parts per Million : Carbon13

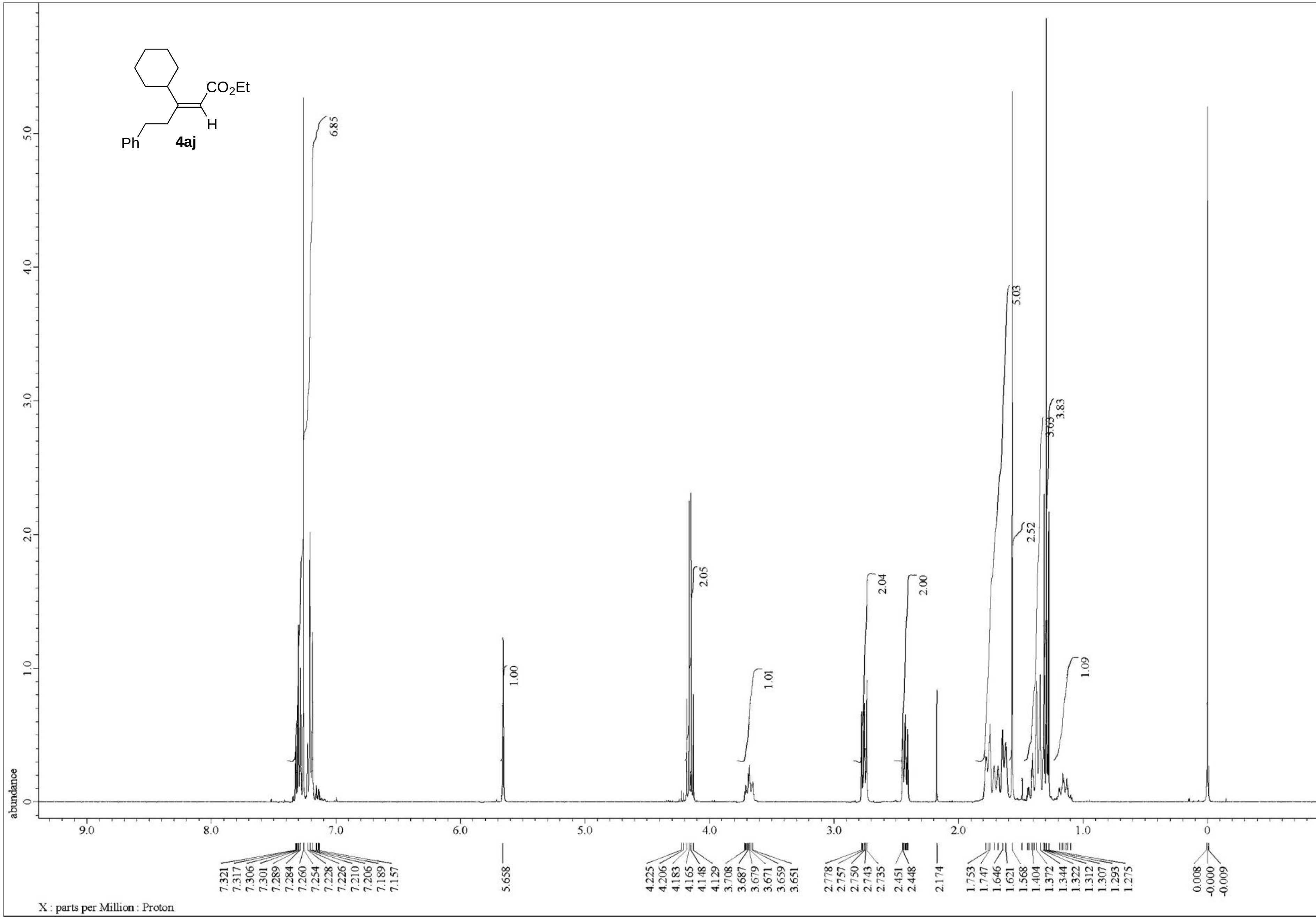
CO2Et
H
Ph
4aj
abundance
X : parts per Million : Proton

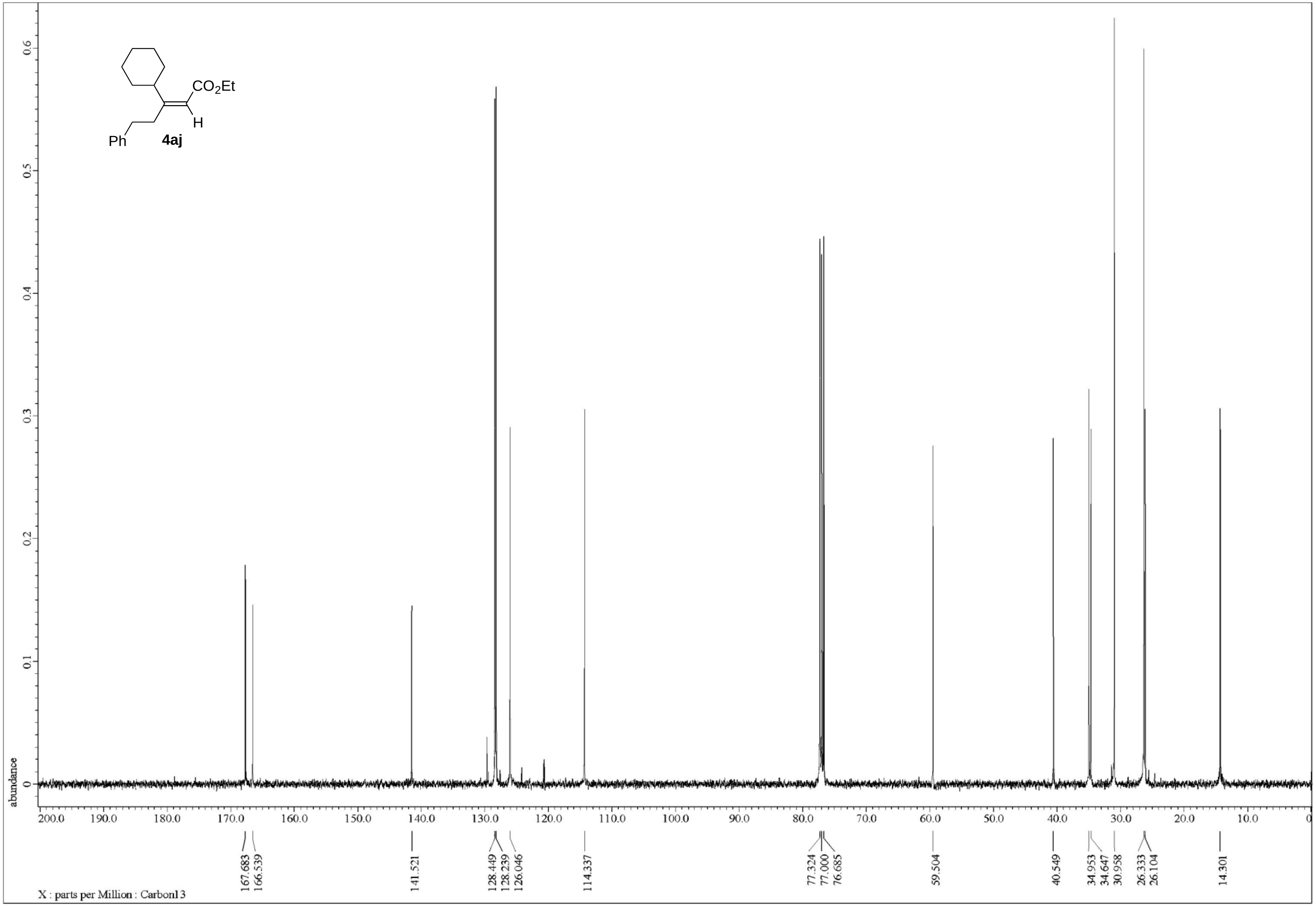
CO2Et
H
Ph
4aj
abundance
0.6
0.5
0.4
0.3
0.2
0.1
0
200.0 190.0 180.0 170.0 160.0 150.0 140.0 130.0 120.0 110.0 100.0 90.0 80.0 70.0 60.0 50.0 40.0 30.0 20.0 10.0 0
167.683
166.539
141.521
128.449
128.239
126.046
114.337
77.324
77.000
76.685
59.504
40.549
34.953
34.647
30.958
26.333
26.104
14.301
X : parts per Million : Carbon13

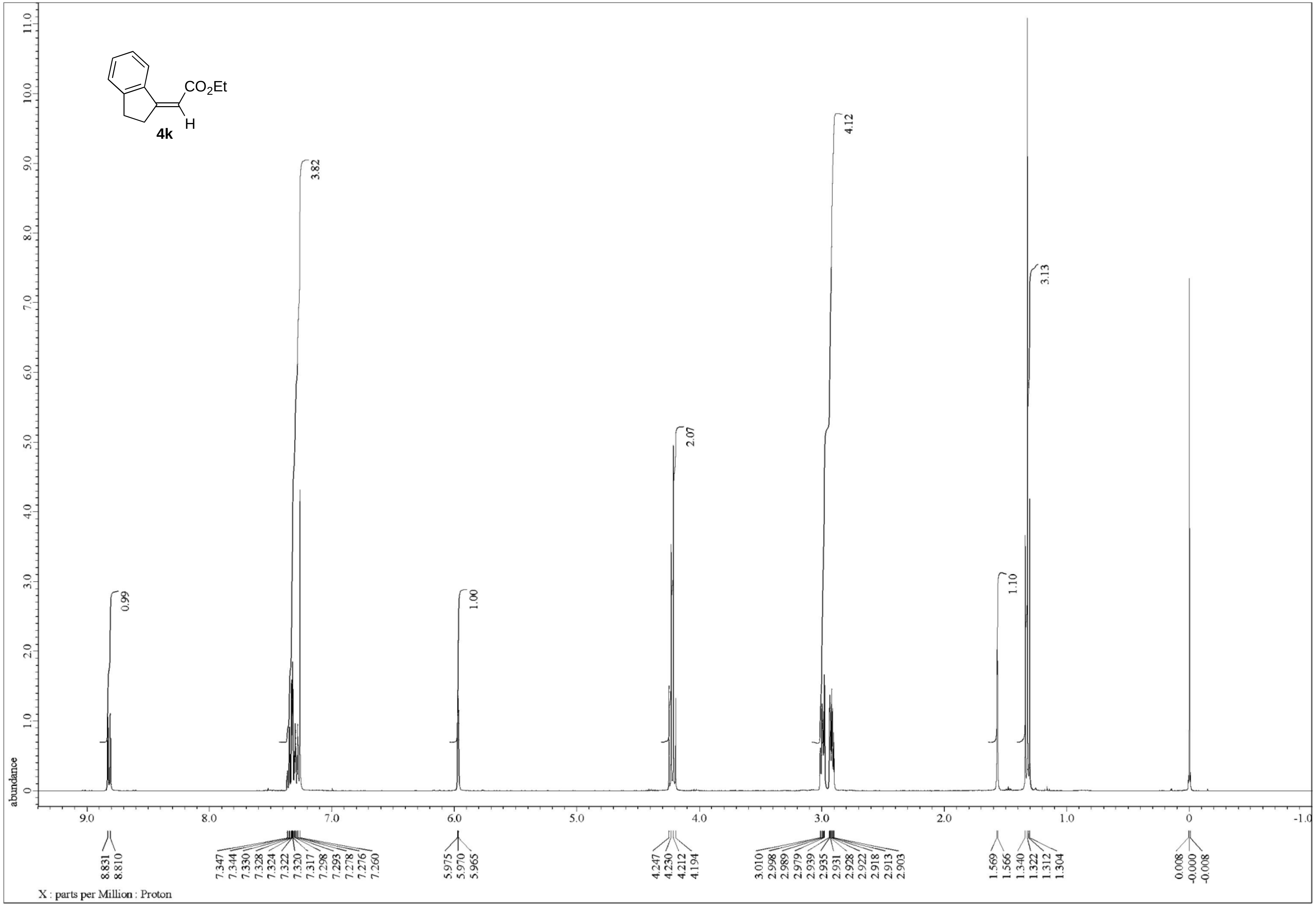

CO2Et
H
4k
abundance
X : parts per Million : Proton
0.99
3.82
1.00
2.07
4.12
1.10
3.13
8.831
8.810
7.347
7.344
7.330
7.328
7.324
7.322
7.320
7.317
7.298
7.293
7.278
7.276
7.260
5.975
5.970
5.965
4.247
4.230
4.212
4.194
3.010
2.998
2.989
2.979
2.939
2.935
2.931
2.928
2.922
2.918
2.913
2.903
1.569
1.566
1.340
1.322
1.312
1.304
0.008
-0.000
-0.008

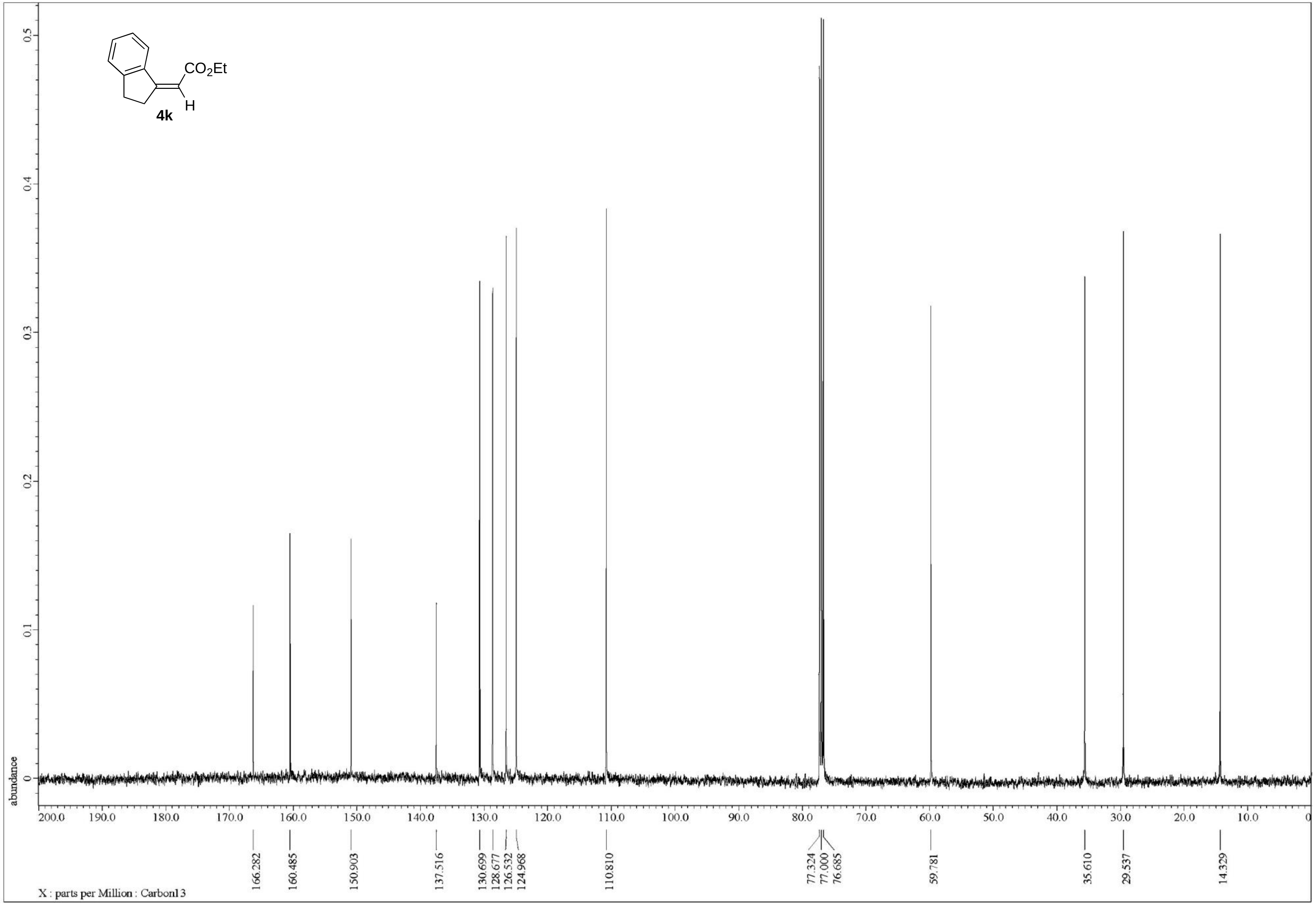
CO2Et
H
4k
abundance
0.5
0.4
0.3
0.2
0.1
0
200.0 190.0 180.0 170.0 160.0 150.0 140.0 130.0 120.0 110.0 100.0 90.0 80.0 70.0 60.0 50.0 40.0 30.0 20.0 10.0 0
166.282
160.485
150.903
137.516
130.699
128.677
126.532
124.968
110.810
77.324
77.000
76.685
59.781
35.610
29.537
14.329
X : parts per Million : Carbon13
